# The Changing Capabilities of Cohorts of the Elderly in Russia during 1990–2020: Measurement using a Quantitative Index

**DOI:** 10.1007/s12062-017-9179-1

**Published:** 2017-05-09

**Authors:** Christopher Mark Davis

**Affiliations:** 1 School of Interdisciplinary Area Studies and Oxford Institute of Population Ageing, University of Oxford, Oxford, UK; 2 Research Laboratory on the Economics of Health Reform, Russian Presidential Academy of the National Economy and Public Administration, Moscow, Russia; 3 Center for Health Economics, Management and Policy (CHEMP), National Research University Higher School of Economics St. Petersburg, St. Petersburg, Russia

**Keywords:** Elderly, Cohorts, Capabilities, Human capital, Russia, Quantitative index

## Abstract

Russia has had a high elderly share of its population like the OECD countries, but has had a more turbulent history over the past 100 years, which has caused fluctuations in the capabilities of those turning 60 (measured by education and training, income, enabling environment, medical care, and health status). This article analyses the life experiences and capabilities of five Russian birth cohorts turning 60 over the period 1990–2020. It presents relevant concepts, reviews past research, and evaluates the importance of health factors (health environment, health-related behaviours, medical care, health status) in determining the activities and contributions of older people in Russia. A Human Capabilities of the Elderly in Russia Index (HCERI) with 22 indicators is developed. Russian data are used in the calculation of the HCERI for the cohorts turning 60 in 1990, 1995, 2000, 2010 and 2020. The article then presents evaluations of the experiences and changes in capabilities for each of the five selected cohorts of the elderly in four periods of life: Childhood (1–15 years), Young Adult (16–49), Mature Adult (50–59), and Early Elderly (60–69). The implications of changes in the characteristics of the elderly for Russian government policies are discussed.

## Introduction

The process of population ageing in the developed countries has been accompanied by advances in educational attainments, professional experiences, income and wealth, and health status (e.g. life expectancy) of the elderly.^1^ Furthermore, societies have improved the ‘enabling environment’ of older people.^2^ These positive developments have made it more possible for the elderly to use their capabilities to make contributions to the economy for wages, to society through volunteer work, and to their households (childcare, education) (Bussolo et al. 2015; Harper 2015).^3^

Russia has an elderly share (19.9% in 2015) above the global average (12.5%), like OECD countries, but has had a more turbulent history over the past 100 years, with adverse changes in several periods in income, societal support, and health of the elderly. It also has had greater inequalities between older citizens. Given these variations, it is illuminating to study the life experiences of Russian birth cohorts turning 60 and their capabilities in older age.^4^

The goals of this article are to answer four related questions:

- *What is an appropriate multi-dimensional quantitative index to measure the evolution over time of the capabilities of the elderly in Russia?*
- *Have the population groups entering old age in Russia from 1990 onwards remained impoverished, poorly educated and unhealthy, or have improvements in their capabilities meant that they can provide substantial benefits to the economy and society, as in leading OECD countries?*
- *How important have health factors (health environment, health-related behaviours, medical care, health status) been in determining the activities and contributions of the elderly in Russia?*
- *What are the implications of projected population ageing and changes in the capabilities of the elderly for the policies of the Russian government out to 2020?*

The second section presents relevant definitions, concepts and measures: birth cohorts (for the years 1930, 1935, 1940, 1950 and 1960 in Russia) and population age-gender pyramids; life experiences of cohorts from birth to the status of elderly; human capabilities and human capital of the elderly; inequalities related to older people and pensions; and production of health of the elderly at the household and national level. It then critically reviews three existing indexes of population well-being and describes the alternative *Human Capabilities of the Elderly in Russia Index* (*HCER Index*, or *HCERI*) that has been developed for this article. The structure, specific indicators, and relevant data of the *HCERI* are described in a sub-section.

The article’s empirical analysis is contained in the third section and readers more interested in results than methodology are encouraged to proceed to it after this introduction. The initial sub-section explains the calculation of the *HCER Index* and shows its aggregate values for the years when the selected birth cohorts reach 60 (in parentheses) and the measures of the five Component sub-indexes: 1990 (45.7), 1995 (27.6), 2000 (30.8), 2010 (52.4), and 2020 (58.6). It then evaluates the life experiences (Appendix Tables 10, 11, 12, 13 and 14) and changes in capabilities (education and training, income, enabling environment, medical care and health status) (Tables 4, 5, 6, 7 and 8) for the five selected cohorts of the elderly in four periods of life: Childhood (1–15 years), Young Adult (16–49), Mature Adult (50–59), and Early Elderly (60–69).^5^ The fourth section generalizes from the analysis to compare changes in the human capabilities of the elderly in Russia in the sub-periods of 1960–1990, 1991–1999, 2000–2016 and 2017–220. This is followed by conclusions.

## Concepts and Measures Concerning Human Capabilities of the Elderly

### Birth Cohorts and Gender-Age Pyramids in Russia: 1989 and 2015

From 1930 to 1990 the size of the population of Russia (RSFSR and Russian Federation) increased from 100.4 million to 148.0 million, while the number those aged 60 and older rose from 8.2 million (8.2% of the population) to 22.5 million (15.3%) (Davis 2006). The male share of the elderly declined from 34% in 1955 to 31% in 1990 due to higher male age-specific death rates. The age group 60–69 contained 57% of elderly in 1990.

**Fig. 1:**
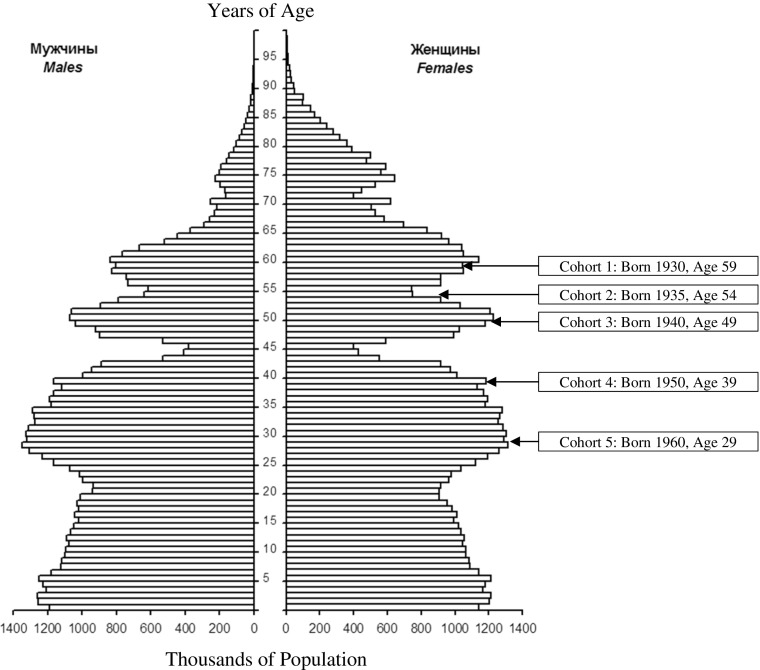
Russia age-gender population pyramid in 1989 and five birth cohorts of the elderly. Source: The population pyramid is from GKRFS *Demograficheskii* 2015, 28. The author drew the five cohort boxes.

Fig. 1 illustrates the relationships of the five birth cohorts with the Russian age-gender population distribution in 1989 (a census year).^6^ The pyramid shows that there were substantially fewer men than women in age groups 45 years and above, a category that contained birth cohorts 1930, 1935 and 1940. For example, in 1989 at the age of 60 there were 861,000 men and 1,176,000 women.

These disparities were caused both by life experiences (collectivization, purges, World War II) and the destructive health-related behaviours of men (excessive consumption of alcohol, smoking) (see sub-sections in third section). The more even balances between the genders for cohorts 1950 and 1960 reflected their lives in peaceful circumstances with rising living standards.

The severe political, economic and social problems in the 1990s caused falling births rates and rising mortality rates (Ellman 2000; Shkolnikov and Cornia 2000; Davis 2001a). However, the size of the population remained stable in the range of 147–148 million because substantial inward migration from former-USSR states offset negative natural growth (births minus deaths).^7^ The ageing process continued, with the share of elderly rising from 15.8% in 1990 to 18.1% in 1999 (Davis 2006). The number of women per 100 men aged 60 and above decreased from 224 in 1989 to 189 in 2002. The numbers and shares of the population in the older (70+ years) age groups continued to increase.

The size of the population in Russia declined from 146.6 million in 2000 to a low of 142.7 in 2009, but then recovered to 146.5 in 2016 (GKRFS 2016 *Rossiiskii*). The number of the elderly (60 and older) rose from 26.9 million in 2000 to 29.1 million in 2015, while their population share increased from 18.5% to 19.9%. The elderly remained predominantly female, but the number of women per 100 men dropped to 185.

**Fig. 2:**
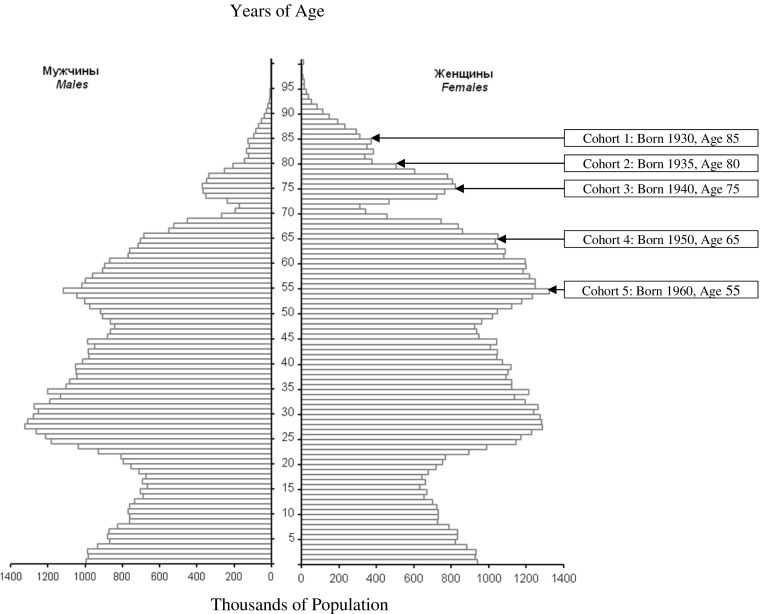
Russia age-gender population pyramid in 2015 and five birth cohorts of the elderly. The population pyramid is from GKRFS *Demograficheskii* 2015, 31. The author drew the five cohort boxes.

The gender-age population pyramid in 2015 with the five birth cohorts is shown in Fig. 2. By that year there were relatively few survivors from the cohorts of 1930 (396,000), 1935 (527,000) and 1940 (1,187,000). There was approximate gender balance for age groups below 55 years, but substantial differences for older population groups. For example, the ratio of women to men for birth cohort 1930 at age 85 was 3.5, whereas for birth cohort 1960 at age 55 it was 1.2.

### Projections of the Population, Economy and Human Capabilities out to 2020

Over the period since 1992 conflicting forecasts of the Russian population have been made by a variety of government organisations (Russia State Statistical Agency, U.S. Bureau of the Census), international bodies (U.N., World Bank), think tanks, and individual scholars (Eberstadt & Groth 2010). In the 1990s many of these predicted substantial declines in the future, whereas in reality the size of the population of Russia in 2016 was slightly lower that it was in 1991.^8^ Due to the mixed record of forecasters, this article has made use of the 2015 official Russian government projections (three variants) of a total population in 2020 in the range of 146.9 million to 148.8 million, which are consistent with those of the World Bank (GKRFS 2015 *Demograficheskii*, pg. 237). The middle variant anticipates a rise in the population older than the working age (women 55, men 60) from 35.2 million in 2015 to 38.7 million in 2020 (or by 10%), which suggests that the total number aged 60 and above will increase to 32.1 million, or 21.7% of the total population. The male share of the elderly should continue to recover, so the gender-age pyramid in Russia should become more symmetrical, even above the age of 60.

The author has based the projections of the economy and human capabilities out to 2020 (see sub-sections on the 1960 birth cohort in the third section) on four major forecasting documents: (1) the 2012 900-page *Strategiya* 2020 prepared by a Russian joint academic-government Expert Group, which contains chapters on the economy, health and pensions (*Strategiya 2020 *2012); (2) the May 2016 forecast of the Russia economy out to 2019 by the Ministry of Economic Development (REF MER 2016); (3) the November 2016 medium-term forecasts (out to 2018) of the Russia economy presented in World Bank (2016); and (4) preliminary material from the 2017 report entitled *Strategy for Russia in 2018–2024* being prepared by the Centre for Strategic Research under Alexei Kudrin (*Strategiya Rossii *2017). The first document has been revealed to be over-optimistic because it was based on a forecast price of oil of $100 per barrel, whereas the early 2017 price is around $50﻿. But the latter three are consistent in presenting baseline forecasts that anticipate moderate oil prices and slow growth of the economy (around 1.8% per annum), living standards and pensions from 2017 onwards.

### Human Capabilities of Cohorts of the Elderly

There are alternative conceptualizations concerning the endowments of individuals, or groupings of them, and their abilities to contribute to the quality of their lives and to society and the economy: human capabilities and human capital. Although the analysis in this article primarily reflects capabilities concepts, some of the insights of human capital theory have been incorporated.

The capabilities approach has been elaborated from ideas of Sen (1979, 1985) and explores how individuals make use of endowments and freedoms to choose paths to achieve multi-dimensional outcomes they value.^9^ It provides insights into the evaluation of well-being over a lifetime, which makes it suitable for this cohort-based study of the elderly. Table 1 presents information about the five components of human capabilities: education and professional training/experience (EW); income security (IN); enabling environment (EE); medical care (MC); and health status (HS). For each component the table provides a description and information about its production, capital, individual contributions (agency), and contributions by society and government.

**Table 1:** Features of components of human capabilities of the elderly

Characteristics of human capabilities	Components of human capability				
	Education	Work training	Income	Enabling	Health status
Descriptions of human capabilities	Capability to use knowledge and skills from education to continue to contribute to the economy, society and family	Capability to use skills from work experience and training to continue to contribute to the economy, society and family	Capability to use the support of income from employment and pensions to continue to contribute to the economy, society and family	Capability to use the support of social capital, housing, social and medical care, security, transport and political engagement to continue to contribute to the economy, society and family	Capability to benefit from full or restricted health to continue to contribute to the economy, society and family
Production of human capabilities	Education system provides teaching and assessment, individuals learn, qualifications awarded	Work experience and training offer skills, individuals learn, qualifications awarded	Post-retirement employment and pensions based on past work provide income	Enabling environment institutions provide opportunities/services to assist ﻿individuals	Health production based on individual behaviour and the medical system reduce illness, disability and mortality
Capital of human capabilities	Education buildings and staff	Training facilities and staff	Availability of jobs and pensions (state, joint, individual)	Social networks, housing, social and medical care facilities/staff, police, transportation, political system	Health stock determined by genetic inheritances, environmental factors, and personal behaviour
Individual contributions to human capabilities	Decisions to continue with non-compulsory education and degree of engagement	Decisions to participate in work training and degree of engagement	Decisions to contribute to pensions and to continue employment after retirement	Decisions to maintain social networks, arrange housing, use social and medical care, avoid crime risks, engage in politics	Decisions to pursue positive health behaviours and avoid negative ones
Government and society contributions to human capabilities	Provide investment and resources to develop and support education system	Provide investment and resources to develop and support work training	Provide investment and resources to maintain employment opportunities and provide pensions to eligible elderly	Provide investment and resources to develop and support housing, social and medical care, police, public transportation, political engagement	Provide investment and resources to maintain a healthy environment and to encourage positive health behaviours

Sources: Prepared by the author on the basis of concepts and information contained in UNDP (2013) and HelpAge International (2013).

The capabilities of the elderly are strongly influenced by the acquisition throughout their lifetimes of formal education and professional training/experiences in employment (EW). Older people who are better educated and have professional skills may find it easier to continue in white-collar employment and to make contributions through volunteer activities than those with lower educational attainment and more limited technical skills. By the age of 60 most people will have acquired all their education and professional skills, although additional training can make positive marginal contributions.

Income security (IN) of the elderly describes the capacity to obtain sufficient income to meet needs in older age and to live independently. If income security is effective, then the elderly can make contributions outside the sphere of formal employment, such as through voluntary work. In contrast, inadequate income results in unwanted dependence, deprivation, and stress.

In order to live independent and fulfilling lives older people require an enabling environment (EE) that has multiple dimensions: (1) access to medical care; (2) existence of social capital (e.g. support networks) (OECD 2010); (3) provision of social care (e.g. nursing homes); (4) protection against criminal activity; (5) access to public transport; (6) housing; and (7) political participation. In this article, medical care (MC) is treated separately from EE in the empirical measurement of capabilities in order to sharpen the focus on the health production of the elderly.

Maintenance of a positive health status (HS) is a key determinant of the abilities of individuals to make contributions to society and the economy. Since the risks of serious illness, disability and death inevitably rise in old age, improvements in health-related behaviours and provision of effective medical care are needed to enable the elderly to remain healthy enough to utilize their education and skills.

The traditional concept of ‘human capital’ refers to the stock of competencies/skills, knowledge, and personal attributes (e.g. creativity) that enable people to make contributions to their family, society and the economy (e.g. provide labour in return for wages) (Becker 1975). The stock of human capital depreciates over time, but this can be offset by investments, which reflect resource allocation decisions of individuals and governments that generate returns. Broader conceptions of human capital recognise that its components include not only education, skills, and experience components (EW), but also income security (IN), the enabling environment (EE), and health status (HS) (Burton-Jones and Spender 2011).^10^ As is evident, concepts of human capital have evolved over the years to resemble those of human capabilities.

Human Capabilities (HC) can be expressed by the following functional relationship:

1 $$ HC= f\left( EW, IN, EE, MC, HS\right) $$

In the advanced OECD countries, both the HC of the elderly and its components usually have changed incrementally in a positive manner over time (e.g. $\frac{\Delta EW}{\Delta t}>0$), although there have been some adverse impacts generated by exogenous economic shocks (e.g. the 2007–09 Global Financial Crisis). However, Russia (within the USSR and independent) has experienced considerable instability and declines in HC, IN, EE, MC and HS at different times over the period since 1990.

### Inequalities Related to Birth Cohorts and the Elderly in Aggregate

Although members of birth cohorts share common political, economic and social experiences throughout their lives, by the time they reach 60 they have differing human capabilities, which reflect their education, employment, gender, health-related behaviours, family status, and geographic location (urban-rural, region). Although this article makes use of average indicators of capabilities of birth cohorts, it should be recognised that this is a measure of the central tendency across the distribution (like the mode and median). Some people have good health, comfortable pensions, high standards of education and skills, and strong support networks, whereas others experience a ‘lived reality’ that involves poverty, illness and isolation. The degree of dispersion of human capabilities varies between birth cohorts and time periods (perhaps less during they socio-economic crisis of the 1990s when most of the elderly population experienced worsening conditions).

The total elderly age group in a given calendar year is made up of members of birth cohorts from 60 years upwards to around 100 years, with each cohort having its unique profile of inequalities. The distribution of a characteristic, such a pension provision, for the whole set of the elderly or a sub-set such as pensioners, is an aggregation of the 40 cohort distributions. The inequalities in these distributions, and especially the difficult circumstances of those at the lower end, should be remembered when reading the empirical third section, even if the text does not always discuss this explicitly due to space constraints.

### Human Capabilities and Production of Health of the Elderly

**Fig. 3:**
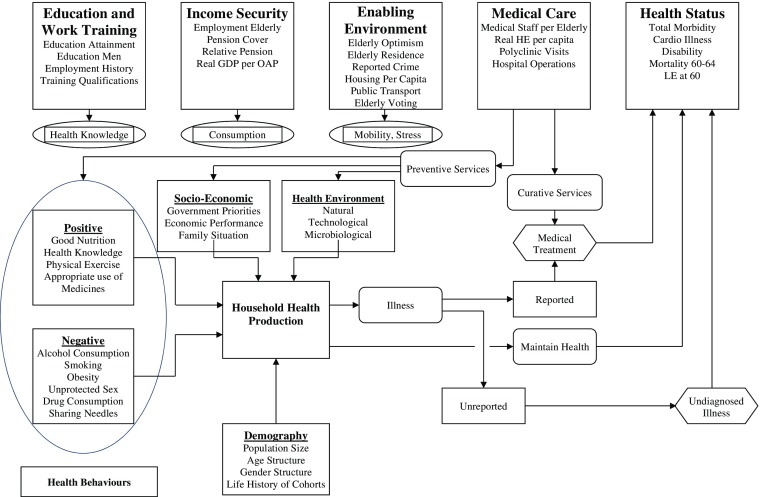
Health in the Human Capabilities of the Elderly in Russia Index. Source: The figure was prepared by the Author on the basis of a diagram of the health production process published in Davis (2001a).

Due to the increasing importance of the dynamics of health status in older age relative to changes in other capabilities, this section explores in more detail health production of the elderly at the household and sectoral level, which are described in Fig. 3 (Auster et al. 1972; Davis 1988, 2001a; Bolin 2011). Demographic, socio-economic and health environment factors influence the health ‘stock’ of members of the population that reflects age, gender, genetic inheritance, and the life experiences of cohorts. Household health production in the current period also is strongly influenced by past and current health-related behaviours (Zweifel and Breyer 1997, Chapter 3; Grossman 1999; Folland et al. 2013). Positive ones include proper diet, exercise, acquisition of health knowledge, timely visits to doctors, and correct use of medicines. Detrimental behaviors include excessive consumption of alcohol and drugs, smoking, poor diet resulting in obesity, unprotected sex, and sharing of needles during injection of drugs. Preventive medical care can offset many of the negative influences on the health of the elderly.

The outcomes of household health production are continuation of good health or onset of illness. Most illness is reported to the medical system, but some remains hidden (reported illness plus hidden illness make up the ‘morbidity iceberg’) (Bhopal 2012, 172; Davis 2017a). Curative medical services are produced by medical facilities and personnel and involve the diagnosis and treatment of illnesses to generate recovery to full health and to minimise the outcomes of invalidity and death (Folland et al. 2013). Illnesses that have been hidden and are revealed at late stages have worse outcomes than do those reported in a timely manner.

Fig. 3 shows that Medical Care (MC), one component of capabilities of the elderly, plays an important role in generating Health Status (HS), another component. The latter is a major determinant of the ability of older people to continue to participate in social, economic and political life making use of the accumulated education, skills and income. Health status can be measured by indicators of maintenance of and recovery to full health, reported and hidden illness, chronic illness, invalidity, and mortality.

### Measurement of Human Capabilities Using Quantitative Indexes

In order to develop empirical measures of the HC of cohorts of the elderly an examination was made of three indexes that measure either human capabilities or human capital in countries of the world on a comparative basis: the UNDP *Human Development Index* (*HDI*) for the total population (UNDP 2013); the World Economic Forum *Human Capital Index* (*HCI*) for the total population (World Economic Forum 2013); and the *Global AgeWatch Index* (*GAWI*) for the elderly (HelpAge International 2013).^11^ This review led to the creation by the author of a new *Human Capabilities of the Elderly in Russia Index* (*HCERI*), which has an appropriate design for the comparison of the capabilities of the five cohorts of the elderly in Russia from 1990 to 2020.

#### UNDP Human Development Index (HDI) of the General Population

The United Nations Development Program (UNDP) has developed a multi-dimensional index to measure ‘human development’ based on capabilities concepts (see footnote 9), which is published in its annual *Human Development Report* (UNDP 1990...^2015^). The *HDI* includes measures of three of the Components of HC (but calls them Dimensions): *Longevity* (HS, measured by Life Expectancy at Birth), *Knowledge* (part of EW, measured by Mean Years of Schooling and Expected Years of Schooling), and *Living Standards* (IN, measured by Gross National Income per capita in US$ at Purchasing Power Parity).

These indicators are transformed if necessary so that they generate positive numbers with ascending values. Minimum and maximum values are established for each indicator on the basis of international experiences, which enables the range to be normalized so that its values vary between 0 and 1. The three Dimension sub-indexes are calculated by using either the simple formula shown in footnote 12 (for HS, IN) or the Geometric Mean (see section two) for the two EW indicators.^12^ The aggregate index is calculated as the Geometric Mean of the Dimension sub-indexes. In 2013 the HDI was calculated for 187 countries.

The UNDP has been critical of the concept of human capital, which it describes using its original narrow interpretation.^13^ However, the *HDI* is more limited in its coverage than both of the alternative indexes discussed below, in that they take into account the Enabling Environment (EE) of people (e.g. social capital) and use more indicators.

#### World Economic Forum (WEF) Human Capital Index (HCI) of the General Population

The *HCI* is explicitly based on theories, models and measurements of Human Capital, broadly defined (World Economic Forum 2013). It has four Components (called Dimensions) with associated statistical indicators: (1) *Education* (part of EW, measured by indicators of quantitative and qualitative aspects of education); (2) *Health and Wellness* (HS, measured by indicators of the population’s physical and mental well–being); (3) *Workforce and Employment* (part of EW, measured by indicators of the experience, knowledge and training of the working–age population); and (4) *Enabling Environment* (EE, measured by indicators of the legal framework, infrastructure and other factors that enhance returns on human capital (see Table 4 of World Economic Forum 2013)). Although the *WEF HCI* is a weighted sum of sub-indexes of its different Dimensions, as with the *HDI*, the former is different from the latter (and the *GAWI* presented in the next section) with respect to its selection of Dimensions, indicators forming sub-indexes, and the statistical method of combining the indexes. For example, the *HCI* is based on 51 indicators, whereas the *HDI* makes use of four (World Economic Forum 2013; UNDP 2013).

In order to standardize the measurements of its indicators, which use differing scales, the *HCI* uses Z–scores (instead of the geometric mean as in the *HDI*), which are calculated from the distributions of the data related to an indicator across the countries under study (122 in 2013).^14^ The values of the four Dimension sub-indexes are calculated as the unweighted averages of its indicator scores. The *HCI* for the total population of a country is determined by multiplying the four Dimension scores by equal weights of 0.25 and adding them.

#### Global AgeWatch Index (GAWI) of the Wellbeing of the Elderly

The *GAWI* 2013 is the first aggregate index of the social and economic well-being of older people that has been calculated on a comparable basis for over 90 countries of the world (HelpAge International 2013, 2014a). In conceptual terms, the index is an offspring of the UNDP *HDI* in that it attempts to measure human development (including capabilities) and outcomes, rather than human capital and inputs. However, the *GAWI* has a structure with respect to Components (called Dimensions) and indicators, that is more similar to the *WEF HCI* than to the *HDI*. Given that the former is designed explicitly to measure human capital, it is justifiable to interpret the *GAWI* as a hybrid empirical measurement of the HC of the elderly.

The *GAWI* takes into account 13 indicators in the four Dimensions of *HC* discussed above (EW, IN, HS, EE). The EW Dimension uses two indicators: (1) *Employment of older people* and (2) *Educational status of older people*. The Dimension IN contains four indicators: (1) *Pension income coverage*; (2) *Poverty rate in old age*; (3) *Relative welfare of older people*; and (4) *Gross domestic product (GDP)* per capita. Dimension HS is measured using three indicators: (1) *Life expectancy at age 60*; (2) *Healthy life expectancy at 60*; and (3) *Psychological wellbeing*. The EE Dimension contains four indicators: (1) *Social connections*; (2) *Physical safety*; (3) *Civic freedom*; and (4) *Access to public transport*. However, it does not measure medical care or social care for the elderly. Furthermore, most of the EE indicators are based on subjective (qualitative) assessments by the elderly obtained from contemporary *Gallup WorldView* surveys, rather than on official statistics.

The methodology used in constructing the GAWI is similar to that of the HDI in its initial steps: (1) convert indicators so they have positive numbers with ascending values, and (2) normalize the values to form a measure in the ranges of 0 to 1 (HelpAge International 2014b). However, in step (3) the *GAWI* calculates the Dimension sub-indexes using the *Weighted Geometric Mean* of their indicators, which involves finding the product of the normalized indicator values raised to the power of their weights (see formula in section two). Step (4) calculates the aggregate country *GAWI* index using the unweighted *Geometric Mean* of the 4 Dimension sub-indexes.

#### Human Capabilities of the Elderly in Russia Index (HCERI)

The three indexes reviewed above offer ideas about the structure and calculation methods that would be appropriate for an index of the capabilities of the elderly in Russia, measured over time instead of internationally. With respect to Components (Dimensions), the *GAWI* appears to be most relevant because it was designed to assess the circumstance of older people. But the selections of indicators of sub-indexes of Components need to be altered for Russia due to reasons of data availability, completeness of coverage, and appropriateness. The data issue relates to the facts that Russia has not published necessary statistics (1990, 1995, 2000, 2010) for numerous ideal indicators and that the Gallup surveys used in the *GAWI* were not conducted for the initial three birth cohort years related to the proposed Russia index. With respect to coverage, the *GAWI* does not contain measures of either social care or medical care, which an index of the capabilities of the elderly should have because both are crucial determinants of active lives. The appropriateness issue relates to the heavy reliance of the *GAWI* in the EE Dimension on surveys of opinion, rather than physical/financial indicators based on official government statistics.

Taking all these factors into account, this paper makes use of the new *HCERI*, which has the five Components (EW, IN, EE, MC, HS) shown in Eq. 1, with some indicators in each of them different from those in the *GAWI*. Although the *HCERI* considers MC to be an element of EE in conceptual terms, it separates it out as an independent Component in the index to emphasize the importance of medical care for the elderly.

The methodology of constructing the aggregate index follows that of the *GAWI*. The initial two steps involve data adjustment and normalization. The *HCERI* calculates the five Component sub-indexes using the *Weighted Geometric Mean* (*a*_1_ , *a*_2_ …  , *a*_5_) of their indicators, which involves finding the product of the normalized indicator values (*X* = {*x*_1_, *x*_2_…, *x*_*n*_}) raised to the power of their weights (*W* = {*w*_1_, *w*_2_…, *w*_*n*_}):

$$ W e i g h t e d\kern0.3em  G e o m e t r i c\kern0.3em  M e a n\kern0.3em \left({a}_i\right)={\left(\prod_{i=1}^n{x}_i^{w_i}\right)}^{\raisebox{1ex}{$1$}\!\left/ \!\raisebox{-1ex}{${\sum}_{i=1}^n{w}_i$}\right.}= \exp \left(\frac{\sum_{i=1}^n{w}_i \ln {x}_i}{\sum_{i=1}^n{w}_{i.}}\right) $$

The aggregate *HCERI* is calculated as the *Geometric Mean* of the five Component sub-indexes (a_1..._a_5_) by finding the *n*th root of product of their values:

$$ Geometric\  Mean={\left({\prod}_{i=1}^n{a}_i\right)}^{\raisebox{1ex}{$1$}\!\left/ \!\raisebox{-1ex}{$ n$}\right.}=\sqrt[ n]{a_1{a}_2\dots {a}_n} $$

### *HCERI* Indicators and Russian Statistical Data

The structure of the *HCER Index* with its five Components was determined on conceptual grounds as discussed above. However, the indicators used in actual calculations of the index for the years in which the birth cohorts reached 60 reflected choices made both in advance and after the examination of data. Due to the fact that the purpose of the *HCER Index* was to measure the capabilities of different birth cohorts of the elderly in one country over a thirty-year period, rather to compare characteristics of the elderly in a contemporary time period across numerous countries, indicators needed to be chosen that could be measured by available Russian data, primarily official statistics.

In the first instance this involved examining the statistical yearbooks of the USSR (GKS SSSR *Narodnoe Khozyaistvo SSSR*, *Okhrana Zdorov’ya v SSSR*, and *Sotsial’noe Razvitie SSSR*) for the years 1989–91 and the Russian Federation (GKRFS *Demograficheskii Ezhegodnik Rossii*, *Meditsinskoe Obsluzhivanie Naseleniya Rossiiskoi Federatsii*, *Rossiiskii Statisticheskii Ezhegodnik*, *Rossiya v Tsifrakh*, *Sotsial’noe Polozhenie i Uroven’ Zhizni Naseleniya Rossii*, *Zdravookhranenie v Rossiiskoi Federatsii: Statisticheskii Sbornik*) for the years 1992–2016. In addition, material from publications of Russian non-governmental institutions, Western governments, and international organizations was used (e.g. US CIR 1993, World Bank 2007, 2015b). The data collection effort produced a database covering the period 1990–2015 with complete or partial entries for 148 variables. Data were available for all years for some of the targeted indicators that had been used in the UNDP *HDI*, WEF *HCI* and HelpAge *GAWI*. But there were insufficient data for some initially specified indicators. As a result, alternatives were selected that had more complete statistical records. In all cases, the substitute indicators measured aspects of capabilities that are similar to the intended ones. With respect to projections out to 2020 for the birth cohort 1960, use was made of material from Russian demographic forecasts and the four studies identified in the beginning of section two.

The final selection of the 22 indicators by Component Sub-Index and their values for the five years are shown in Table 2. Many of the measures are obvious (*1.1 Educational Attainment*) and do not require explanation, but others have less straightforward meanings or may appear to duplicate coverage within a Component. Brief comments are provided below to explain or justify selected indicators.

**Table 2:** Human Capabilities of the Elderly in Russia Index: Structure and indicator values for 1990, 1995, 2000, 2010, 2020

			Years				
	Measure	Sources	1990	1995	Years 2000	2010	2020
**1. Education and employment indicator values**							
1.1 Educational Attainment of Population Aged 60–64	Higher & Intermediate per 1000	GKS, GKRFS	194	224	407	600	646
1.2 Working OAPs as share of total OAPs	%	GKS, GKRFS	23.5	26.4	16.1	34.9	38.0
1.3 OAP Aged 60–72 as share of total employment	%	GKS, GKRFS	4.2	3.0	4.0	4.2	5.0
**2. Income security indicator values**							
2.1 Pension coverage	%	GKS, GKRFS	91.0	96.1	96.4	100.0	100.0
2.2 Old age pension as a share of the average wage	%	GKS, GKRFS	39.9	54.7	40.2	40.4	45.0
2.3 OAP as share of subsistence minimum	%	GKS, GKRFS	237	99	76	165	200
2.4 Real GDP per Old age pensioner index	1990 = 100.0	GKS, GKRFS	396.4	229.8	2442	367.6	477.9
**3. Enabling environment indicator values**							
3.1 Social capital index of happiness Gen population	% Happy	VTsIOM	39	40	44	52	48
3.2 Social care: residence beds	Number	GKS, GKRFS	225	221	223	249	2(50
3.3 Public safety. reported crime of theft	Number	GKS, GKRFS	913.1	1367.9	1310.1	1108.4	1100.0
3.4 Housing: urban living space	Sq M per capita	GKS, GKRFS	16.4	18.1	19.2	22.6	24.0
3.5 Public transportation	Passenger KM-millions	GKS, GKRFS	791	552	496.2	483.8	450
3.6 Political participation: share elderly voting x share voting for ruling party	%	CSPP	SO	50	60	70	65
**4. Medical care indicator values**							
4.1 Medical Staff Per Elderly Person	Number	GKS, GKRFS	108.0	92.9	83.4	86.9	88.0
4.2 Real Health Expenditure Index	1990 = 100	GKS, GKRFS	100.0	74.7	73.7	130.2	156.0
4.3 Capacity of Polyclinics	Visits per Shift	GKS, GKRFS	21.7	23.6	24.5	25.8	27.0
4.4 Medical Operations in Hospitals	Number	GKS, GKRFS	62.4	57.1	59.0	64.9	68.0
**5. Health Status Indicator Values**							
5.1 Total Population Morbidity (Incidence) Rate	Cases per 1000	GKS, GKRFS	651.2	676.0	730.5	780.0	790.0
5.2 Cardiovascular Morbidity (Incidence) Rate	Cases per 1000	GKS, GKRFS	11.2	13.2	17.1	26.1	28.0
5.3 Disability (Incidence)	Cases per 1000	GKS, GKRFS	5.2	9.1	7.7	7.7	8.0
5.4 Mortality Rate of Age Group 60–64 Years	Deaths per 1000	GKS, GKRFS	34.2	47.1	45.0	37.1	34.0
5.5 Life Expectancy at 60	Years	GKS, GKRFS	14.7	13.1	13.2	14.6	16.0

Note on Sources: The statistics in this table were collected by the author as described in the text. 20 of the 22 indicators are based on information contained in official statistics (those from GKS and GKRFS). The sources of the other two were CSPP (2016) and VTsIOM (2015).

Within the Income (IN) Component *2.3 Old Age Pension as a Share of the Subsistence Minimum* helps to measure poverty of the elderly, whereas *2.2 Old Age Pension as a Share of the Average Wage* reflects the priority that the government awards the elderly, with the share rising as priority increases, such as in the 2000s. The indicator *2.4 Real GDP per Old Age Pensioner Index* provides a summary of the resources that society has available to allocate to various needs. It is useful in the Russian context because of the variation in real GDP growth: a 40% drop in the 1990s, rapid recovery (8% growth per year) in the 2000s, downturn during the Global Financial Crisis (2008–09), slow growth during 2010–13, and negative growth again during 2014–16.

Global Age Watch identified *3.5 Public Transportation* as a key component of the Enabling Environment (EE) of the elderly. The included indicator highlights the realities that there has been a substantial decline in provision of public bus and train services during the transition from a socialist/command economy to a market-oriented one and that state subsidies of public travel have been reduced.

The political involvement of the elderly is measured by *3.6 Political Participation: Share Elderly Voting x Share Voting for Ruling Party*. Although the share of elderly voting tends to remain high, the share of elderly voting for the ruling party measures their satisfaction with government policies. In Russia it was low in the mid-1990s and the elderly voted for the reformed Communist Party, whereas it became high in the 2000s when there was greater social stability and rising real pensions.

In the Health Status (HS) Component *5.1 Total Population Morbidity (Incidence) Rate* reflects demographic (age, gender), socio-economic, and health conditions in the society, whereas *5.2 Cardiovascular Morbidity (Incidence) Rate* measures developments in a specific disease that is the most important cause of death of the elderly in Russia. The indicator *5.4 Mortality of Age Group 60–64 Years* is an outcome measure related to the largest five-year age group of the elderly, which has responded quickly to changing socio-economic conditions. In contrast, *5.5 Life Expectancy at 60* estimates the expected number of years that a person at 60 will live on the assumption that the age-specific mortality rates in the year of measurement remain constant over time.

## Measurement Using the *HCER Index* of the Changing Capabilities of Birth Cohorts Turning 60 in Russia during 1990 to 2020

### Calculation of the *HCER Index* for 1990, 1995, 2000, 2010 and 2020

The calculations of the *HCER Index* and its sub-indexes were made using an Excel spreadsheet adapted by the author from one provided to him by HelpAge International, which it developed for the *GAWI*.^15^ The *HCERI* worksheet has six sections (five Capabilities Component Sub-Indexes and the Aggregate Index). For each Component Sub-Index the data for each indicator was provided by year (shown in Table 2), with the values for 2020 representing projections based on best available evidence (including official government estimates). These data were adjusted so that rising values meant improving capabilities. This was necessary only for indicators of crime and mortality, both of which increased (deteriorated) in the 1990s. The indicators then were normalized relative to low and high values to generate measures in the 0.0 to 100.0 range.^16^ The next step involved assigning to the indicators weights that totalled 10 across all indicators for a Component (integers were necessary for the Excel formula). The weights reflected the author’s assessment of the relative importance of indicators within a Component (e.g. Life Expectancy at 60 was given a higher weight than the Disability Rate).^17^ Appendix Table 15 contains the normalising standards and weights of the 22 indicators. The Sub-Indexes then were calculated using the *Weighted Geometric Mean* formula. The five Component Sub-Indexes were combined into the aggregate *HCER Index* using an unweighted geometric mean.

The calculated total *HCER Index* and Component Sub-Indexes for each birth cohort in the year it reached 60 are presented in Table 3. The first column shows that the value of the *HCERI* was 45.7 in 1990 (index value in Column 2 = 100.0). It dropped to a low of 27.6 (60.4) in 1995 and then recovered to 30.8 (67.4) in 2000 due to improvements in EW, EE and HS. The *HCERI* value rose substantially to 52.4 (114.7) in 2010 because of significant positive changes in all of the components. The upward trend of the *HCERI* is projected to continue through to 2020, when it should reach 58.6 (or 28.2% above the 1990 value). The reasons for these developments in the *HCERI* are discussed in detail in the five sub-sections below.

**Table 3:** Calculations of HCER Index total and by components: 1990, 1995, 2000, 2010, 2020

Year	**HCER index value**		**I. Education and employment index value**		**II. Income security index value**		**III. Enabling environment index value**		**IV. Medical care index value**		**V. Health status index value**	
1990	45.7	100.0	23.4	100.0	59.8	100.0	52.3	100.0	42.0	100.0	64.8	100.0
1995	27.6	60.4	24.3	103.8	33.3	55.7	29.9	57.2	33.4	79.5	19.9	30.7
2000	30.8	67.4	32.7	139.7	27.8	46.5	32.5	62.1	31.8	75.7	29.7	45.8
2010	52.4	114.7	64.8	276.9	58.2	97.3	47.9	91.6	53.1	126.4	41.3	63.7
2020	58.6	128.2	74.6	318.8	76.8	128.4	44.9	85.9	62.2	148.1	43.1	66.5
2010/1990	1.15		2.77		0.97		0.92		1.26		0.64	
2020/1990	1.28		3.19		1.28		0.86		1.48		0.67	

The EW, IS, EE, MC and HS sub-indexes were calculated by the Author as weighted geometric means of the values that are presented in Table 2. The aggregate HCER Index was calculated as the unweighted geometric mean of the five sub-index values. The weights used in the calculations are shown in Table 15 in the Appendix.

### The Birth Cohort of 1930 Turning 60 in 1990 in the RSFSR

#### Experiences during Four Phases of Life: 1930–1990^18^

The birth cohort of 1930 had an extremely challenging childhood (Appendix Table 10), with family strains caused by the Stalin dictatorship, rapid industrialization, forced collectivization, and World War II. Children lived in poverty in over-crowded housing with poor diets and limited facilities (e.g. sports, catering, libraries) in under-funded schools. Although economic and social conditions improved gradually after the war, living standards remained low until the mid-1950s. In subsequent years the political system became less repressive and the economy grew at high but decelerating rates. However, the health environment worsened due to increasing pollution and self-destructive health-related behaviours of adults (poor diet, heavy alcohol consumption, smoking, inadequate exercise), especially of men, that became entrenched in the population. When this cohort was aged 50–59 it lived in an improving situation until the end of the *perestroika* era (Davis 1987, 1993, 2001a). However, its first decade in the elderly category (1990–99) coincided with the political break-up of the USSR, prolonged negative economic growth, high inflation, increases in inequality and poverty, and cuts in real state budget expenditures. There was a growth in psycho-social stress of the elderly population, which intensified adverse health behaviours.

#### Evolution through Life Phases of the Capabilities of the Birth Cohort of 1930

The evolutions of the capabilities of the cohort that was born in 1930 through life phases are shown in Table 4. In brief, they were adversely affected by its experiences during 1930–1950, but influenced positively by developments over the next 40 years.

**Table 4:** Influences on capabilities of the Russia birth cohort of 1930 turning 60 in 1990

**Category**	**Childhood (1–15): 1930–1944**	**Young Adult (16–49): 1945–1979**	**Mature Adult (50–59): 1980–1989**	**Early elderly (60–69): 1990–1999**
**Education**	System: Rapid expansion of urban schools, low quality, disruption by war.	System: Sustained expansions of schools and universities, quality improves.		System: Limited facilities for education of elderly.
	Results: Rises in literacy and primary/secondary education.	Results: Elimination of illiteracy, rising levels of primary, secondary and university qualifications.		Results: Low levels of educational attainment for Cohort of 1930.
**Employment**	NA	Socialist economy has compulsory full employment. Female participation near demographic maximum.		Virtually all members of Cohort of 1930 have full employment record.
**Training**	NA	Expansion of apprenticeships and on-the-job training. Technical skills of employed population rise continuously.		Cohort of 1930 acquired skills and work experience, but much of its knowledge is outmoded by 1990s.
**Income security**	NA	Social security system initially covers employees of government, industry and state farms. Extended to collective farmers in 1970s.		Most of Cohort of 1930 had state pensions, but real value of them fell in 1990s. Loss of savings. Decline of elderly employment.
**Enabling environment**	Social capital: 1930–53 severe disruption; 1954–89 stable networks of families and friends.			Severe disruption of professional networks.
	Social care: Negligible state facilities, dependent on families.			Negligible availability of state facilities.
	Physical security: 1930–53: insecurity due to dislocation, war; 1954–89 protection against crime.			Breakdown in in order, increases in crimes.
	Housing: 1930–53: severe deficiencies; 1954–89: improved supply, rising per capita provision.			Elderly become owners as sitting tenants.
	Public transportation: rapid growth of rail, bus, metro, tram systems, low cost of public travel.			Cut-backs in provision, increases in fares.
	Political participation: 1930–56: repressive political system; 1957–89 relaxation of controls, adults participate in elections due to compulsion and patriotism.			High participation of elderly in new Russia democracy, but many vote for Communists.
**Medical care**	Expansion of basic medical care, infants and children have high priority.	Substantial expansion of national health service, but it functions as low priority sector in shortage economy. Many doctors and hospital beds, but backward technologies and low quality of treatment.		Real health spending cut by 33%. Reductions in availability and quality of medical care.
**Health status**	High rates of infectious diseases, invalidity, mortality.	Declining rates of infectious diseases and mortality until mid-1960s. Then increases in non-communicable diseases and age-specific death rates until mid-1980s. Anti-alcohol campaign from 1985 reduces mortality temporarily.		Disruptive systemic changes in 1990s result in increases in morbidity and mortality rates of the elderly and delclines in life expectancy at 60.

Sources: This table and the related text in this section are based on extensive research carried out by the Author that involved examination of Soviet and Russia data and literature on the USSR and Russia by academics, governments and international organizations. Due to space constraints references are made primarily to the author’s lectures at Oxford University and publications (Davis 1983a, b, 1987, 1988, 1990, 1993, 2001a, b, 2006, 2015) that contain detailed citations of sources

This cohort benefited from the rapid expansion of primary and secondary education in the 1930s and the elimination of illiteracy. However, disruptions caused by the war held back the education of teenagers and young adults. Overall, the educational standards achieved by this cohort were higher than those of previous generations, but below subsequent ones. In 1990 people aged 60–64 had a rate of middle and higher education of 194 per 1000. The shift to a full employment economy meant that almost all members of the population worked continuously and benefited from expanding on-the-job training. In 1990 the share of old age pensioners (OAPs) who were working was 23.5% and OAPs in the year group 60–72 made up 4.2% of the total workforce. The value of the EW sub-index was 23.4 in 1990.

The social security system (including pensions) was extended to industrial and government workers under Stalin and later to agricultural workers (Madison 1988; Powell 1991). By 1990 91% of the population retiring (women at 55 and men at 60) received old age pensions. Among the main features of Soviet pensions were: their values varied between occupational groups, so there were significant inequalities; the average value of pensions was below the average wage (39.9% in 1990); and the average pension was well above the subsistence minimum (237%). Despite these achievements, many elderly citizens lived in poverty or in difficult circumstances. The value of the IN sub-index was 59.8.

The enabling environment remained severely deficient throughout the Stalin period, but then improved until the end of the USSR, although there was variation across its elements. On average, the new elderly in 1990 had well-developed family and social networks (social capital), adequate protection against crime, good access to public transportation, and satisfactory housing. But as mentioned in section two, inequalities existed in all areas, which meant that many had a ‘lived reality’ that was substantially worse than the average. Social care in the USSR was underdeveloped with respect to the provision of both social workers and specialised residential care. This meant that most disabled or vulnerable older people needed to obtain care from their families. The majority of the elderly identified with the Soviet political system and participated in the highly constrained elections that appointed members of the legislature (the Soviets) at different levels. The value of the EE sub-index was 52.3.

The elderly in the USSR had access to a comprehensive national health service that provided medical care free of direct charge. The quality of preventive and curative services was low up to the 1960s, but standards were raised gradually after that (Davis 1983b; 1987; 1993). Large inequalities existed because medical care was provided through six sub-systems (elite, ministerial, industrial, large cities, medium cities, and rural) with varying standards (Davis 1988). The value of the MC sub-index was 42.0.

The health status of the cohort of 1930 improved relative to low Russian historical standards during 1950–65: infant mortality declined and life expectancy at 60 increased. But this was followed by two decades of rising age-specific death rates and falls in life expectancy, which reflected the lagged impacts of stressful life experiences, poor diet, lack of exercise, excessive consumption of cigarettes and alcohol, and inadequate responses to growing non-communicable disease by the insufficiently-funded medical system (Davis and Feshbach 1980). Health outcomes improved from 1985 to 1990, in part because of the anti-alcohol campaign of the Gorbachev regime (Ellman 1994; Eberstadt & Groth 2010). The 1990 value of the HS sub-index was 64.8.

The multi-dimensional weighted *HCER Index* has taken these contradictory tendencies into account (e.g. improving education and medical care, but worsening health status) and has a calculated value of 45.7. It is likely that the index value for the cohort of 1930 in 1990 was higher than equivalents for the cohorts of 1910 and 1920 when they turned 60, but it is not possible to carry out a rigorous comparison due to data limitations. The main role of the 1990 index is to establish a baseline for comparison of subsequent measures in Russia for the years 1995, 2000, 2010 and 2020.

Table 4 indicates that the circumstances of members of the cohort of 1930 changed substantially during their initial decade in the elderly status (1990–99). Although the value of sub-index EW continued to rise, those of IN, EE, MC and HS deteriorated. In consequence, it is almost certain that the overall measure of the capabilities of this cohort worsened in the 1990s. However, by the time this cohort reached 70 the Russian economy was recovering and they experienced a decade of improvements in all components of capabilities.

### The Birth Cohort of 1935 Turning 60 in 1995 in the Russian Federation

#### Experiences during four Phases of Life: 1935–1995

Although the birth cohort of 1935 is close to that of 1930 in calendar terms and both had similar adverse experiences in childhood, the former was chosen for inclusion in this study because it illustrates clearly the impacts on the capabilities of the elderly of negative developments in the economy and society in later years (see Appendix Table 11). After a difficult childhood period, the young adults of the 1935 cohort benefited from the post-Stalin years of rising affluence. But the growing prevalence of negative health-related behaviours, especially among men, undermined their health status, which was reflected in rising mortality after 1965. This cohort turned 50 years at the start of the Gorbachev-led *perestroika* period, which was one of ambitious reforms, growing economic and political disruptions and social disorientation (Aslund 1991; Brown 1996; Ellman and Kontorovich 1992). The second half of their fifties and beginning of their sixties were spent in the difficult initial transition decade involving the break-up of the USSR, the severe ‘transformational recession’ (output decline) in the early 1990s, and the financial crisis of 1998. However, there were substantial improvements in the economic and social spheres and in the health environment from 2000 onwards.

#### Evolution through Life Phases of the Capabilities of the Birth Cohort of 1935

The educational standards of the 1935 cohort improved relative to the past because of the greater participation of its members in education (Table 5). The indicator of middle and higher education per 1000 aged 60–64 rose from 194 in 1990 to 224 in 1995. As young adults they were fully employed in an expanding economy and the professional training they received was of an improving standard. However, many of the skills they acquired in the command economy were not relevant to the evolving market economy of the 1990s, which made it difficult for them to adapt to new circumstances in the labour market. By 1995 the share of working old age pensioners had increased to 26.4%, but the share of those aged 60–72 in the total workforce had declined to 3.0%. Many low income elderly people were forced to work in in the unregulated ‘second economy’ for meagre rewards. The value of the EW sub-index remained roughly stable at 24.3 in 1995.

**Table 5:** Influences on capabilities of the Russia birth cohort of 1935 turning 60 in 1995

Category	Childhood (1–15): 1935–1949	Young Adult (16–49): 1950-1984 Mature Adult (50–59): 1985–1994	Mature Adult (50–59): 1985–1994	Early Elderly (60–69): 1995–2004
**Education**	System: Rapid expansion of urban schools, low quality, disruption by war.	System: 1950–91: Sustained expansions of schools and universities, quality improves; 1992–94 cuts in budget spending, numerous reforms, deterioration in quality.		System: 1995–99 Limited and deteriorating facilities for education of elderly; 2000–04 improvements in adult education.
	Results: Rises in literacy and primary/secondary education.	Results: 1950–94: Elimination of illiteracy, rising levels of primary, secondary and university qualifications.		Results: Low levels of educational attainment for 1935 Cohort due to early experiences.
**Employment**	NA	1950–91: Full employment of men and women in socialist economy; 1992–94 unemployment increases in malfunctioning market economy.		Virtually all members of Cohort of 1935 had full employment records.
**Training**	NA	1950–91: Expansion of apprenticeships and on-the-job training. Technical skills of employed population rise; 1992–94 deterioration of training.		Cohort of 1935 acquired skills and work experience, but much of its knowledge is outmoded in Russia market economy.
**Income security**	NA	Social security system initially covers employees of government, industry and state farms. Extended to collective farmers in 1970s. Coverage continued in Russia period.		Most of Cohort of 1935 had state pensions, but real value of them fell after 1991. Loss of savings. Decline of elderly employment.
**Enabling environment**	Social capital: 1935–53 severe disruption; 1954–91 stable networks; 1992–94 deterioration.			1995–99 worsening; 2000–04 improvements.
	Social care: Negligible state facilities, dependent on families.			Negligible availability of state facilities.
	Physical security: 1935–53: insecurity; 1954–89 protection against crime; 1990–94 worsening.			1995–99 worsening; 2000–04 improvements.
	Housing: 1935–53: severe deficiencies; 1954–94: improved supply, rising per capita provision.			Elderly become owners as sitting tenants.
	Public transportation: 1935–91 rapid growth, low cost; 1992–94 cuts in services and rising costs.			Cut-backs in provision, increases in fares.
	Political participation: 1935–56: repressive political system; 1957–91 adults participate in elections; 1992–94 participation declines and older population votes against reformist government.			1995–99 elderly vote for Communists; 2000–04 high participation, elderly vote for Putin.
**Medical care**	Expansion of basic medical care, infants and children have high priority.	1950–91 Expansion of health service, but it functions as low priority sector in shortage economy. Many doctors and hospital beds, but backward technologies and low quality of treatment; 1992–94: cuts in health spending and deterioration in medical care.		1995–99 Cuts in real health spending, worsening of medical care; 2000–04 increases in health spending and improvements in services.
**Health status**	High rates of infectious diseases, invalidity, mortality.	1950–64 declining rates of infectious diseases and mortality; 1965–84 increases in non-communicable diseases and age-specific death rates; 1985–88 reductions in mortality from anti-alcohol campaign; 1989–94 increases in mortality, declines in life expectancy.		1995–2003 increases in morbidity and mortality rates of the elderly, declines in life expectancy at 60; 2004 mortality falls.

Sources: This table and the related text in this section are based on extensive research carried out by the Author that involved examination of Soviet and Russia data and literature on the USSR and Russia by academics, governments and international organizations. Due to space constraints references are made primarily to the author’s lectures at Oxford University and publications (Davis 1983b, 1987, 1988, 1993, 2001a, b, 2006, 2015) that contain detailed citations of sources

This cohort continued to benefit from improvements in the social security system. By 1995 96.1% of the retiring population was eligible for a work-related and state-financed old age pension. The average pension as a share of the average wage rose to 54.7% in 1995, but its share of the subsistence minimum dropped from 237% to 99%. The real value of pensions fell in the nineties and the index of real GDP per OAP fell from 396 in 1990 to 230 in 1995. Living conditions were even worse than the mean for those at the lower end of the pension distribution. Many more of the elderly in Russia experienced poverty. The older population lost most of their carefully accumulated life savings in the early 1990s due to high inflation and the lack of indexing protection. By 1995 the value of the IN sub-index had fallen to 33.3, from 59.8 in 1990.

There were significant changes, mostly adverse, in the enabling environment that affected the 1935 cohort at the age of 60. The intensifying break-up of the extended family and rising bankruptcies and unemployment undermined traditional social capital and forced the elderly to establish new survival networks (Round 2006). Although housing continued to be a positive aspect of the life of older people (especially due to the privatization of their flats in city centres), they were adversely affected by rising crime, curtailment of public transportation and real increases in its prices, and worsening of already inadequate social care. Many of the elderly became alienated from the government of President Yeltsin and voted for the reformed Communist Party, which called for maintenance of features of the Soviet welfare system. The value of the EE sub-index dropped from 52.3 to 29.9.

This cohort had access to better medical care from about 1965 (at the age of 30) to 1990 (age of 55). Although the national health service remained under-resourced by OECD standards (around 3% of GDP), it expanded in quantitative terms (doctors, middle medical personnel, hospital beds) and there were improvements in medical technologies used in diagnostics and treatment (Davis 1983b, 1987, 1993, 2001a, b). But in the decade of the 1990s there was a 33% cut in real health spending, health reforms proved ineffective, and the quality of medical care deteriorated. Due to these changes, the value of the MC sub-index fell from 42.0 in 1990 to 33.4 in 1995.

The health status of the 1935 cohort improved during 1985–90 due to positive developments during *perestroika* (notably, the anti-alcohol campaign), but then worsened considerably in the 1990s due to the combination of the accumulated impacts of past destructive health-related behaviours, economic collapse, poverty, deteriorating medical care, and rising psycho-social stress (Ellman 1994; Shapiro 1995; Shkolnikov and Cornia 2000; Eberstadt & Groth 2010). Negative trends can be observed in most health outcome indicators during 1990 to 1995: the total illness rate rose from 651 to 676 cases per 1000; the incidence of disability climbed from 5.2 to 9.1 cases per 1000; deaths per 1000 in the age group 60–64 went up from 34.2 to 47.1; and life expectancy at 60 years fell from 14.7 to 13.1 years. The HS sub-index reflected these adverse changes and declined from 64.8 to 19.9.

The multi-dimensional weighted *HCER Index* has taken into account the worsening of almost all indicators in its five Components in the nineties. Its value dropped by 40% from 45.7 in 1990 to 27.6 in 1995.

Table 5 indicates that in the economic recovery period after 2000 most measures of the capabilities of the 1935 cohort improved: the real value of pensions rose, health spending increased, there was greater social cohesion, and there were gains in health status (after 2003). As a result the value of the HCERI of this cohort recovered from its low point in the mid-1990s.

### The Birth Cohort of 1940 Turning 60 in 2000 in the Russian Federation

#### Experiences during four Phases of Life: 1940–2000

The life experiences of the birth cohort of 1940, summarized in Appendix Table 12, were more positive than those of previous cohorts. First, less of early childhood was spent in circumstances of acute deprivation and there were stable social and economic conditions (e.g. the Malenkov re-orientation of the economy toward consumption) in teenage years. Second, throughout the Younger Adult period (ages 16–49) the international environment of the USSR remained peaceful, economic growth was positive, and living standards improved. However, the rising affluence in the USSR was accompanied by the intensification of adverse health-related behaviours, primarily of men. Third, this cohort experienced *perestroika (198*5–1991) and early transition (1992–95) at relatively young working ages (respectively 45–51 and 52–55), which enabled many of its members to adapt to the intensifying systemic changes. However, others were adversely affected by unemployment, loss of savings and psycho-social stress. Fourth, this cohort reached 60 in 2000, which was the start of a decade of greater social stability, positive economic growth, and stronger support of the welfare of the population by the government. For example, the most members of the cohort of 1940 were protected from the negative effects of the Global Financial Crisis in 2009 (when they were 69), Overall, the life experiences of this cohort were significantly more positive than were those of the previous two considered.

#### Evolution through Life Phases of the Capabilities of the Birth Cohort of 1940

The educational standards of the 1940 cohort improved relative to the past (Table 6). By 2000 the indicator of middle and higher education had almost doubled from its 1995 level, to 407 per 1000 aged 60–64. The population entered old age with full employment records in a more technologically advanced and reforming command economy, and with higher standards of professional training, with some of it relevant to a market economy. However, the prolonged economic recession of the 1990s caused drops in participation in the economy by older members of the labour force. By 2000 the share of working old age pensioners had decreased to 16.1%, while the share of those aged 60–72 in the total workforce remained around the historic low of 3%. The generally positive developments resulted in the value of the EW sub-index rising from 24.3 in 1995 to 32.7 in 2000.

**Table 6:** Influences on capabilities of the Russia birth cohort of 1940 turning 60 in 2000

**Category**	**Childhood (1–15): 1940–1954**	**Young Adult (16–49): 1955–1989**	**Mature Adult (50–59): 1990–1999**	**Early elderly (60–69): 2000–2009**
**Education**	System: Disruption by war, expansion of schools and universities, low quality.	System: 1955–91 Sustained expansions of schools and universities, quality improves; 1992–99 cuts in budget spending but increased private funding, uneven improvements in quality.		System: Expansion of educational facilities for the elderly.
	Results: Rises in literacy and primary/secondary education.	Results: 1955–99 rising levels of primary, secondary and university qualifications.		Results: Improved levels of educational attainment for 1940 Cohort.
**Employment**	NA	1955–91: full employment of men and women in socialist economy; 1992–99 unemployment increases in malfunctioning market economy.		Virtually all members of Cohort of 1940 have full employment record.
**Training**	NA	1950–91: expansion of apprenticeships and on-the-job training. Technical skills of employed population rise; 1992–99 deterioration of training.		Cohort of 1940 acquired skills and work experience, but much of its knowledge is outmoded in 1990s market economy.
**Income security**	NA	Social security system initially covers employees of government, industry and state farms. Extended to collective farmers in 1970s. Coverage continued in Russia period.		Virtually all of 1940 Cohort had state pensions. Real value of pensions rises in 2000s. Increases in elderly employment.
**Enabling environment**	Social capital: 1940–53 severe disruption; 1954–91 stable networks; 1992–99 deterioration.			Improving social capital of elderly.
	Social care: 1940–1999 negligible state facilities, dependent on families.			Negligible availability of state facilities.
	Physical security: 1940–53: insecurity; 1954–89 protection against crime; 1990–99 worsening.			2000–04 improvements in physical security.
	Housing: 1940–53: severe deficiencies; 1954–99: improved supply, rising per capita provision.			Elderly become owners as sitting tenants.
	Public transportation: 1940–91 rapid growth, low cost; 1992–99 cuts in services and rising costs.			Cut-backs in provision, increases in fares.
	Political participation: 1940–56: repressive political system; 1957–91 adults participate in elections; 1992–99 participation declines and older population votes against reformist government.			2000–09 high participation, elderly vote for Putin.
**Medical care**	Expansion of basic medical care, infants and children have high priority.	1955–91 Expansion of health service, but it functions as low priority sector in shortage economy. Many doctors and hospital beds, but backward technologies and low quality of treatment; 1992–99: cuts in health spending and deterioration in medical care.		2000–09 increases in health spending and improvements in diagnostics and treatment.
**Health status**	High rates of infectious diseases, invalidity, mortality.	1955–64 declining rates of infectious diseases and mortality; 1965–84 increases in non-communicable diseases and age-specific death rates; 1985–88 reductions in mortality from anti-alcohol campaign; 1989–99 increases in mortality, declines in life expectancy.		2000–03 increases in morbidity and mortality, declines in life expectancy at 60; 2004–09 mortality falls, LE at 60 rises.

Sources: This table and the related text in this section are based on extensive research carried out by the Author that involved examination of Soviet and Russia data and literature on the USSR and Russia by academics, governments and international organizations. Due to space constraints references are made primarily to the author’s lectures at Oxford University and publications (Davis 1983b, 1987, 1988, 1993, 2001a, b, Davis 2006, 2015) that contain detailed citations of sources

The income situation of the new elderly in 2000 was affected by lagged negative impacts of the 1990s and insufficient benefits from the increase of the index of real GDP per OAP from 230 to 244. The share of those retiring who received an old age pension remained unchanged at 96%. Although the real value of the old age pension increased by 28% in 2000, this was from a low base. The OAP share of the average wage fell back to the 1990 value of around 40% and the average OAP as a share of the subsistence minimum fell further to 76%. A significant number of this cohort in the lower end of the pension distribution lived in dire circumstances, although the shares of the elderly living in poverty slightly decreased from 1995 to 2000: women aged 55 and older from 21.1% to 19.6% and men aged 60 and older from 17.1% to 15.3%. These somewhat mixed changes resulted in the value of the IN sub-index decreasing from 33.3 to 27.8.

The enabling environment of the 1940 cohort in 2000 improved only slightly from that of the 1935 cohort in 1995. Social capital was enriched due to greater stability in society. This was reflected in a rise in the Index of Happiness from 40 to 44. There also were small increases in indicators related to social care (e.g. residence beds), public safety (e.g. thefts down from 1.4 million to 1.3 million), housing (e.g. an increase from 18.1 to 19.2 m of living space per capita), and political participation of the elderly. However, provision of public transportation continued to decline, from 552 passenger km-millions in 1995 to 496 in 2000. The combined impacts of these changes resulted in the value of the EE sub-index increasing modestly from 29.9 to 32.5.

Developments in medical care were similar to those in EE in general because the positive changes in the economy did not immediately benefit the national health service. The number of medical staff per elderly person fell from 92.9 in 1995 to 83.4 in 2000. The index of real health expenditure remained unchanged. With respect to medical service provision, the capacity of polyclinics increased slightly, as did the number of medical operations in hospitals. But large inequalities in access to higher quality medical care persisted. These offsetting trends caused the MC sub-index to fall from 33.4 to 31.8.

The 1940 cohort avoided the problem of rising age-specific death rates from 1965 to 1985 because they were too young to be affected (25–45). Health outcomes improved slightly during 1985–90 but then deteriorated significantly in the 1990s. By 2000 morbidity rates were higher than in 1995, the disability rate and the age-specific death rate for the age group 60–64 were lower, and life expectancy at 60 remained stable. The net effect of these developments was that the HS sub-index increased from 19.9 to 29.7, which was still less than one-half of its value of 64.8 in 1990.

The overall influences on this cohort of changes related to capabilities in Russia from 1995 to 2000 were modestly positive. This is reflected in the small increase in the *HCER Index* from 27.6 to 30.8. But that value was substantially lower than that of 1990 (45.7)

The final column of Table 6 indicates that the better Life Experiences in Russia in the 2000s, identified in Appendix Table 12, resulted in substantial improvements in the capabilities of the 1940 cohort during its initial decade in the status of elderly. With respect to income, both real pensions and employment rose. There were advances in the enabling environment and the standard of medical care was raised due to greater real health spending. Although inequalities remained problematic in all sphere, there were reductions of dispersions around rising means.

### The Birth Cohort of 1950 Turning 60 in 2010 in the Russian Federation

#### Experiences during Four Phases of Life: 1950–2010

The birth cohort of 1950 had more positive life experiences than those of the three previous cohorts examined in this article (Appendix Table 13). Its childhood years were spent in peace with a recovering economy and a political leadership that was more committed to living standards than the Stalin regime. During the initial twenty years of Young Adult life the mature Soviet political system maintained stability and the economy grew at positive but decelerating rates. Both personal and collective consumption increased substantially. This generation was too young to be afflicted by the rising age-specific death rates during 1965–85. Members of this cohort were only 25 at the start of *perestroika* and 42 when the shifts to democracy and the market economy occurred, so they were able to engage in the reforms more effectively than older generations and take advantage of the new opportunities. Their Mature Adult years (50–59) were spent in a rapidly growing economy with a stable political system. There were improvements in both the health environment and individual health behaviours (e.g. more modest alcohol consumption and reduced smoking). Although the Global Financial Crisis adversely affected the Russian economy in 2009, the government was able to protect the population by drawing on its large stabilization fund. So this cohort was in a strong position when it reached 60 years in 2010.

As indicated in Appendix Table 13, over its initial six years in the elderly category the cohort of 1950 has been affected by the slower growth of the economy, a new recession in 2014–16 caused by dramatically lower oil prices, and greater international tensions linked to the Ukraine and Syria crises (Davis 2016, 2017b). The government has not possessed the resources to protect citizens fully from the economic crisis, so many of the elderly have experienced falling living standards since 2013.

#### Evolution through Life Phases of the Capabilities of the Birth Cohort of 1950

During 1950–80 the USSR significantly expanded its higher education system and improved the quality of primary, secondary and tertiary education. As a result, there was a decisive improvement in the educational standards of the 1950 cohort (Table 7). The indicator of middle and higher education rose from 407 per 1000 aged 60–64 in 2000 to 600 in 2010.

**Table 7:** Influences on capabilities of the Russia birth cohort of 1950 turning 60 in 2010

**Category**	**Childhood (1–15): 1950–1964**	**Young adult (16–49): 1965–1999**	**Mature adult (50–59): 2000–2009**	**Early elderly (60–69): 2010–2019**
**Education**	System: expansion of schools and universities, quality improves in 1960s.	System: 1965–91 expansions of schools and universities, quality improves; 1992–99 cuts in budget spending but increased private, unsuccessful reforms; 2000–09 increased funding and quality.		System: Improvements in educational facilities for the elderly.
	Results: Higher attainments in primary/secondary education.	Results: 1965–2009 rising levels of primary, secondary and university qualifications, with disruptions in 1990s.		Results: Significantly improved levels of educational attainment for 1960 Cohort.
**Employment**	NA	1965–91: full employment in socialist economy; 1992–99 higher unemployment; 2000–09 close to full employment.		Most members of 1960 Cohort have full pension qualifying employment record.
**Training**	NA	1965–91: expansion of apprenticeships and on-the-job training. Technical skills of employed population rise; 1992–99 deterioration of training; 2000–09 improvement in skills training for market economy.		1960 Cohort acquires skills and work experience relevant to demands of market economy.
**Income security**	NA	1965–2009: Social security system initially covers employees of government, industry and state farms. Extended to collective farmers in 1970s. Coverage continued in Russia period.		Most of 1960 Cohort has state pensions, but gender/class differences. Rising real value of pensions. Increases in elderly employment.
**Enabling environment**	Social capital: 1960–91 stable; 1992–99 deterioration; 2000–19 improved.			Improving social capital of elderly.
	Social care: 1960–2015 negligible state provision; 2016–2019 expansion of state and private facilities.			Greater availability of state and private care.
	Physical security: 1960–53: insecurity; 1954–89 protection; 1990–99 worsening; 2000–19 improving.			Improvements in physical security.
	Housing: 1960–53: severe deficiencies; 1954–2019: improved supply, rising per capita provision.			Most elderly owners, but rising homelessness.
	Public transportation: 1950–91 rapid growth, low cost; 1992–2009 cuts in services and rising costs.			Cut-backs in provision, increases in fares.
	Political participation: 1950–56: repressive political system; 1957–91 adults participate in elections; 1992–99 participation declines, more votes for opposition; 2000–09 popular support for Putin.			2010–18 high participation of elderly, who vote for Putin.
**Medical care**	Improvements in medical care, infants and children have high priority.	1965–91 Expansion of health service, but it is a low priority sector in shortage economy with backward technologies; 1992–99: cuts in health spending, worsening medical care; 2000–09 increases in health spending and improved medical care.		2010–19 increases in health spending and improvements in diagnostics and treatment for the elderly.
**Health status**	Decreasing rates of most infectious diseases for children but rising infant mortality.	1965–84 increases in non-communicable diseases and age-specific death rates; 1985–88 reductions in mortality from anti-alcohol campaign; 1989–2003 increases 2004–09 morbidity increases, mortality falls, life expectancy rises.		2010–19 high morbidity, declines in mortality, LE at 60 rises.

Sources: This table and the related text in this section are based on extensive research carried out by the Author that involved examination of Soviet and Russia data and literature on the USSR and Russia by academics, governments and international organizations. Due to space constraints references are made primarily to the author’s lectures at Oxford University and publications (Davis 1983b, 1987, 1988, 1993, 2001a, b, 2006, 2015) that contain detailed citations of sources

Most of the adult population had worked full-time throughout their lives and had benefited from professional training during their careers. Their more modern work skills enabled them to engage more fully with a competitive market economy. Due to the economic circumstances in Russia from 2000 onwards and the higher quality of labour of this cohort, the share of working OAPs doubled to 34.9% in 2010 and the share of 60–72 year-old workers in the labour force rose to the 1990 level of 4.2%. In consequence, the value of the EW sub-index doubled from 2000 to 64.8.

The income situation of the elderly improved significantly during the 2000s because of both high employment rates and rises in real pensions. By 2010 100% of those retiring were eligible for state pensions. The real value of old age pensions rose every year from 2000 to 2010. The OAP share of the average wage remained around 40%, but its share of the subsistence minimum increased to 165%. The index of real GDP per OAP rose significantly from 244 to 368. The number of the elderly living in poverty decreased markedly. Due to the uniformly positive changes, the value of the IN sub-index increased from 27.8 in 2000 to 58.2 in 2010.

There were significant improvements in five of the six elements of the enabling environment, which substantially enhanced this component of capabilities of the elderly in 2010. Social capital continued to develop positively, with the Index of Happiness rising from 44 to 52. The numbers of beds for social care and of social workers increased. Most categories of crime diminished, with the number of cases of theft declining from 1.3 million to 1.1 million. The indicator of metres of living space per capita rose from 19.2 to 22.6. Political participation of the elderly increased. The only negative development was the contraction of public transportation, with a fall from 496 passenger km-millions in 2000 to 484 in 2010. The value of EE sub-index increased significantly from 32.5 to 47.9.

Over the period 2000–2010 there were improvements in medical care: the number of medical staff per elderly person rose from 83.4 to 86.9; the capacity of polyclinics increased from 24.5 to 25.8 million visits per shift; and the number of medical operations in hospitals went up from 59.0 to 64.9 million. Another positive development was the almost doubling of the index of real health expenditure from 73.7 to 130.2. These changes drove up the MC sub-index from 31.8 to 53.1.

During the 2000s morbidity rates continued to rise, although these increments were caused partially by the population reporting illnesses that previously had been hidden (Davis 2017a). The invalidity rate remained stable. A major improvement with respect to health outcomes was the sustained reduction in all types of mortality rates after 2003. For example, the death rate for the age group 60–64 declined from 45.0 per 1000 in 2000 to 37.1 in 2010. Reflecting this, life expectancy at 60 rose from 13.2 to 14.6 years. These changes resulted in the HS sub-index increasing from 29.7 to 41.3.

The evidence presented above demonstrates that there were positive movements in all five components of capabilities. Due to these consistent changes and the weightings used in the calculations, the *HCER Index* rose from 30.8 in 2000 to 52.4 in 2010. This enabled it to rise above its level at the start of transition in 1990 of 45.7.

The final column of Table 7 indicates that the ‘lived experience’ of the elderly in the cohort of 1950 became more complicated after 2010. The slow growth of the economy during 2010–2013 limited the possibilities of the government of dealing with many existing problems. From 2014 through 2016 the combination of the dramatic fall in world oil prices from $145 per barrel to a low of $28 undermined state budget revenue and resulted in cuts in expenditures in most spheres, including all those related to the elderly. The accompanying depreciation of the ruble resulted in higher inflation, especially for important products like medicines, which over time had become predominantly foreign-sourced (in contrast to prudent self-sufficiency in the Soviet period). Since many of the elderly in Russia were on relatively fixed pensions but prescribed medicines for outpatient treatment were only available at market prices (medicines are free in hospitals), they experienced severe problems in purchasing necessary medical products. However, the ruble exchange rate has appreciated since mid-2016 due to the increases in the world market price of oil, which has been a beneficial development for the elderly. But overall, those in the lower end of the pension income distribution experienced increasing hardship during 2014–2016.

### The Birth Cohort of 1960 Turning 60 in 2020 in the Russian Federation

#### Experiences during Four Phases of Life: 1960–2020

The actual and projected life experiences of the cohort that will turn 60 in 2020 are shown in Appendix Table 14. This generation had generally favourable conditions during Childhood and Young Adult years up to 1990. They experienced the societal disruption and economic collapse of early transition (1992–99) in their thirties, when they were resilient and able to adapt to new circumstances. For most of their forties members of this cohort lived in a stable society with a strong government, worked in an expanding economy, and experienced substantial improvements in living standards. Although the Global Financial Crisis caused negative growth in the economy of Russia in 2009, the government was able to protect the population from its effects by drawing on its accumulated reserve funds.

Russia remained relatively stable politically and socially during the initial phase (2010–2013) of the Mature Adult period of this cohort (years 50–53), but then economic circumstances became more challenging. The economy suffered a recession from mid-2014 through 2016, primarily due to the collapse of the world market price of oil and secondarily to economic sanctions (World Bank 2015a, 2016; Davis 2016, 2017b). The government did not have the financial means to insulate fully the population from economic shocks, so unemployment and poverty increased and average living standards stagnated. However, there were improvements in both the health environment due to tighter pollution controls and health-related behaviours, in that younger adults in urban areas reduced their alcohol consumption and smoking.

Over the next four years (2017–2020) there should be political continuity because there were few substantive changes in the State Duma resulting from the elections in November 2016 (four year terms) and it is anticipated that President Putin will be elected for another six-year term in 2018. Economic forecasts by the Russian government and international organizations (World Bank, IMF) anticipate a recovery of the economy in 2017 from the recession and low positive growth in subsequent years. This should be assisted by a more vigorous economic reform program (being designed by former Finance Minister Alexei Kudrin) and a gradually increasing world market price for oil (up to $50 per barrel). In light of these likely political and economic developments, a reasonable baseline forecast is that there will be only moderate social instability in the period out to 2020. Health-related behaviours should improve over this time due to ongoing energetic and apparently effective anti-smoking and anti-alcohol campaigns by the government.

On the basis of available long-term projections it reasonable to assume that the cohort of 1960 will continue to have largely positive experiences by Russian standards during its initial decade in the elderly status (2020–2029). The economy should be able to maintain steady low growth due to market conditions, prudent fiscal and monetary policies, and deeper economic reforms. However, unless economic and social developments are consistent with the optimistic scenario sketched out above, problems of inequality and poverty could intensify in the 2020s.

#### Evolution through Life Phases of the Capabilities of the Birth Cohort of 1960

Demographic developments in Russia during 2010–2015 and projections to 2020 are evaluated in the second section (see also World Bank 2016). To recap, it is likely that the number of elderly will increase from 24.7 million in 2010 to 29.1 in 2020. There should be improvements in the educational standards of the cohort turning 60 in 2020, reflecting the positive developments in the educational system in earlier years (Table 8) (*Strategiya* 2020, Chapter 10). The indicator of middle and higher education per 1000 aged 60–64 should increase from 600 in 2010 to 646 in 2020.

**Table 8:** Influences on capabilities of the Russia birth cohort of 1960 turning 60 in 2020

**Category**	**Childhood (1–15): 1960–1974**	**Young Adult (16–49): 1975–2009**	**Mature Adult (50–59): 2010–2019**	**Early Elderly (60–69): 2020–2029**
**Education**	System: Expansion of schools and universities, quality improves in 1970s.	System: 1975–91 expansions of schools and universities, quality improves; 1992–99 cuts in budget spending but increased private, unsuccessful reforms; 2000–19 increased funding and quality.		System: Improvements in educational facilities for the elderly.
	Results: Higher attainments in primary/secondary education.	Results: 1975–2019 rising levels of primary, secondary and university qualifications, with disruptions in 1990s.		Results: Significantly improved levels of educational attainment for 1960 Cohort.
**Employment**	NA	1975–91: full employment in socialist economy; 1992–99 and 2013–19 higher unemployment; 2000–12 close to full employment.		Most members of 1960 Cohort have full pension qualifying employment record.
**Training**	NA	1975–91: expansion of apprenticeships and on-the-job training. Technical skills of employed population rise; 1992–99 deterioration of training; 2000–19 improvement in skills training for market economy.		1960 Cohort acquires skills and work experience relevant to demands of market economy.
**Income security**	NA	1975–2019: Social security system covers whole population. Most adults qualify for state pension. Retirement ages rise from 55 for women and 60 for men.		Most of 1960 Cohort has state pensions, but gender/class differences. Rising real value of pensions. Increases in elderly employment.
**Enabling environment**	Social capital: 1960–91 stable; 1992–99 deterioration; 2000–19 improved.			Improving social capital of elderly.
	Social care: 1960–2015 negligible state provision; 2016–2019 expansion of state and private facilities.			Greater availability of state and private care.
	Physical security: 1960–53: insecurity; 1954–89 protection; 1990–99 worsening; 2000–19 improving.			Improvements in physical security.
	Housing: 1960–53: severe deficiencies; 1954–2019: improved supply, rising per capita provision.			Most elderly owners, but rising homelessness.
	Public transportation: 1960–91 rapid growth, low cost; 1992–2019 reduced services and higher costs.			Cut-backs in provision, increases in fares.
	Political participation: 1960–91 adults participate in elections; 1992–99 participation declines, more votes for opposition; 2000–19 popular support for Putin.			2020–29 high participation of elderly, who vote for Putin and successors.
**Medical care**	Improvements in medical care, infants and children have high priority.	1975–91 Expansion of health service, but it is a low priority sector in shortage economy with backward technologies; 1992–99: cuts in health spending, worsening medical care; 2000–09 increases in health spending and improved medical care.		2020–29 increases in health spending and improvements in diagnostics and treatment for the elderly.
**Health status**	Decreasing rates of most infectious diseases for children but rising infant mortality 1971–74.	1975–84 increases in non-communicable diseases and age-specific death rates; 1985–88 reductions in mortality from anti-alcohol campaign; 1989–2003 increases 2004–19 morbidity increases due to ageing, mortality falls, life expectancy rises.		2020–29 high morbidity but lower mortality of new elderly, LE at 60 rises.

Sources: This table and the related text in this section are based on extensive research carried out by the Author that involved examination of Soviet and Russia data and literature on the USSR and Russia by academics, governments and international organizations. Due to space constraints references are made primarily to the author’s lectures at Oxford University and publications (Davis 1983b, 1987, 1988, 1993, 2001a, b, 2006, 2015) that contain detailed citations of sources

By 2020 the professional training of this cohort will have improved relative to earlier ones, especially because its members will have spent their final 30 years of employment in an evolving market economy that is integrated into the global system. It is expected that there will be increases in the shares of OAPs working to 38.0% and of those employed in the age group 60–72 to a peacetime peak for Russia of 5.0%. The combined effects of these positive changes should raise the value of the EW sub-index from 64.8 in 2010 to 74.6 in 2020. (23.4 in 1990).

The income situation of the elderly should continue to improve out to 2020 because of higher participation in the economy by OAPs and increments to real pensions linked to the elections of 2016 and 2018 (*Strategiya* 2020, Chapter 6). The average old age pension is projected to increase to 45% of the average wage due to labour shortages and to 200% of the subsistence minimum (still below the peak of 1990). The index of real GDP per OAP is likely to increase by around 30% from 2010, to 478. Although the number of the elderly living in poverty may decline, many of the elderly will remain in acutely strained circumstances with respect to actual life experiences (inadequate pensions, rising costs of living and medicines, inadequate social care). Taking into account the overall beneficial developments, the IN sub-index is projected to increase from 58.2 to 76.8.

It is likely that there will be mixed changes in the enabling environment out to 2020. Social capital may deteriorate due to stagnant living standards and substantial inequalities. The Index of Happiness is projected to drop from 52 to 48 due to popular dissatisfaction with government policies. There will be other negative changes related to the provision of public transportation (down to 450 millions of passenger kilometers) and political participation (a decline from 70% to 65%). The crime level should remain stable and there should be improvements in social care (more beds and staff) and in housing (living space per capita up to 24.0 square metres). The combined effect of these changes will be to lower the EE sub-index from 47.9 to 44.9.

The MC index in Russia is projected to rise from 53.1 to 62.2. This will be caused by increases in: the number of medical staff per elderly person (to 88.0); real health expenditure (the index will rise to 156); the capacity of polyclinics (to 27.0 million visits per shift); and the number of medical operations in hospitals (to 68.0) (Strategiya 2020, Chapter 13).^19^

On the basis of published evidence concerning developments through 2015 and underlying trends (e.g. population ageing) it is anticipated that cases of reported illness will continue to rise (incidences of total morbidity to 790 cases per 1000 and of cardiovascular disease to 28.0 cases per 1000). However, these indicators are given low weights in the Sub-Index because much of the higher reporting will be generated by the beneficial uncovering of previously hidden illness (Davis 2017a). The invalidity rate probably will rise slightly. It is projected that age-specific mortality rates will continue to decline (e.g. from 37.1 deaths per 1000 to 34.0 for the age group 60–64) and life expectancy will rise (at 60 from 14.6 years to 16.0). Largely as a result of the mortality improvements, the HS sub-index should rise from 41.3 to 43.1.

Projections of changes in the five Component Sub-Indexes out to 2020 indicate that there will be improvements in four (EW, IN, MC and HS) and deterioration in one (EE). If these changes are realized, then the *HCER Index* will increase from 52.4 in 2010 to 58.6 in 2020, which would be 28% above the 1990 value. Although this article does not attempt to forecast specific developments in the capabilities of the 1960 cohort throughout its initial decade in the elderly category, available evidence and projections suggest that in general there will be improvements in them out to 2030. More authoritative projections will be available later in 2017 when the Centre for Strategic Studies publishes its report on *Strategiya Rossii na 2018–2024 gg.*, which has a chapter on Human Capital.

## Comparison of the Capabilities of Cohorts of the Elderly in Russia in Sub-Periods after 1960

As mentioned in the Introduction and demonstrated in the previous empirical section, Russia has experienced substantial variation in the components of human capabilities (EW, IN, EE, MC, HS) of the elderly and in the aggregate measure (HC) compared to international experiences, especially in the OECD countries, where trends have been consistently upward. Table 9 summarises the main developments (positive or negative) in the Components of the capabilities of the elderly in four periods over the years 1960–2020.^20^

**Table 9:** Changes in capabilities of the elderly in Russia by sub-period: 1990–2020

Changes	1960–1990	1991–1999	2000–2016	2017–2020
$\frac{\varDelta HC}{\varDelta EW}$	Positive	Positive	Positive	Positive
$\frac{\varDelta HC}{\varDelta IN}$	1960–88: + ; 1989–90: –	Negative	Positive	Positive
$\frac{\varDelta HC}{\varDelta EE}$	Positive	Negative	Positive	Slight negative
$\frac{\varDelta HC}{\varDelta MC}$	1960–88: + ; 1989–90: –	Negative	Positive	Positive
$\frac{\varDelta HC}{\varDelta HS}$	1960–64: + ; 1965–84: – ; 1985–88: + ; 1989–90: –	Negative	2000–03: – ; 2004–16: +	Positive
$\frac{\varDelta HC}{\varDelta t}$	Low Positive	Negative	Strong Positive	Positive

Sources: The directions of movements in the indicators are based on calculated values of the HCERI for the years 1990–2020 and from past research of the author (see sources below) for the period 1960–1990. This table and the related text in this section four are based on extensive research carried out by the author that involved examination of Soviet and Russia data and literature on the USSR and Russia by academics, governments and international organizations. Due to space constraints references are made primarily to the author’s lectures at Oxford University and publications (Davis 1983b, 1987, 1988, 1993, 2001a, b, 2006, 2015) that contain detailed citations of sources.

Evidence concerning the initial three historical periods confirms that EW has improved uninterruptedly and has exerted a continuous positive influence on HC. Changes in IN (primarily pensions) on average were positive during 1960–88, negative in the period 1989–1999 because of difficulties in the Soviet/Russian economy, and positive again from 2000 onward (with the exception of the recession years of 2014–16). But in each period the inequalities related to the elderly meant that those at the lower end of the distribution lived in difficult circumstances. The aggregate impacts of the enabling environment on capabilities were positive during 1960–1990, negative in the 1990s, and positive again from 2000. Medical care improved in quantitative and qualitative terms during 1960–1989, but its effectiveness was undermined in the 1990s by the accelerating disintegration of the Soviet Union and then by the severe transformational recession, political instability and cuts in real health spending. Preventive and curative medical services improved after 2000, helped by growth of real health expenditures, and therefore had beneficial impacts on the capabilities of the elderly.

The health status of the Soviet elderly population deteriorated during 1965–1984 because of the increases in age-specific death rates and declines in life expectancy at 60 of those years. There was an improvement of HS during the early *perestroika* period, primarily due to the anti-alcohol campaign, but then a sustained worsening over the years 1989–2003. Health status changed in a positive manner during 2004–2016. Although morbidity rates increased during these years, part of this was due to increases in reported illness generated by reductions in hidden illness, which was a beneficial process (Davis 2017a). Overall, the net impacts on the Human Capabilities index of the changes in the five Components shown in Table 9 were Weakly Positive during 1960–1990, Negative during 1991–1999, and Strongly Positive during 2000–2016. However, it should be recognised that even in the periods with Positive changes, the elderly in Russia at the lower end of the income distribution were experiencing a ‘lived reality’ that was difficult by West European standards.

The projected influences on capabilities of the elderly in Russia of changes in the Components over the period 2017–2020 are beneficial for four (EW, IN, MC and HS) and slightly detrimental for one (EE). It is expected that the aggregate impact on HC will be positive.

## Conclusions

The analysis in this article has provided answers to the four questions posed in the Introduction. The second section argued that the concept of ‘capabilities’ should provide the basis for the analysis of the evolution over time of the well-being and abilities of cohorts of the elderly in Russia. Elements of three existing indexes (UNDP *HDI*, WEF *HCI*, HelpAge *GAWI*) were used to construct a new index that is appropriate for the analytical task: the *HCER Index*. It was argued that this index should have five components (EW, IN, EE, MC, HS) and be based primarily on statistical indicators that rely on official Russian government data. The *HCER Index* was calculated for the five years in which the cohorts of 1930, 1935, 1940, 1950 and 1960 turned 60. The changes of the total index and its components are explained in detail in the third section and summarized in the fourth.

The answer to the second question is that the human capabilities of the elderly in Russia have improved substantially from 2000 onward and the newer cohorts of older people have higher educational standards, professional skills and income than their predecessors. Their capabilities have been enhanced by improvements in the enabling environment, medical care and health status. Although an international comparison is beyond the scope of this study, by 2020 the average circumstances and capabilities of the younger elderly in Russia should converge with those in middle-range countries of the OECD region. However, significant inequalities related to the elderly in Russia are likely to continue throughout the next decade.

Health factors have played decisive roles in determining the changes in the capabilities of cohorts of the elderly in Russia. In the Soviet period the polluted health environment, destructive health-related behaviours, and ineffective medical care resulted in rising age-specific mortality of the elderly and falling life expectancy at 60. The health status of older people improved temporarily in the *perestroika* period, but then worsened markedly during 1991–2003. The sustained poor health of elderly people in Russia undermined their ability to make full use of their education and skills to earn extra income through employment beyond the retirement age.

The substantial improvements in the health of elderly cohorts after 2003 have enabled them to make greater contributions to their families, society and the economy. The positive correlation between enhanced health status and greater activity of the elderly in Russia should continue through 2020. Nevertheless, those in the lower socio-economic strata of the elderly will experience relatively worse health outcomes (illness, mortality) than those with higher status.

The answer to the fourth question should recognize that the government in Russia has made energetic efforts improve the situation of the elderly, but has achieved only a record of mixed successes and failures.^21^ On the one hand, it ensures that almost all of the elderly have at least minimal pensions, opportunities to continue part-time employment after retirement, medical care free of direct official charge, and housing. On the other hand, there still are substantial deficiencies in the enabling environment with respect to social care (homes for elderly, support provided by social workers), transportation (cuts in public transport, rising charges), medical care (low quality and hidden informal charges for medical services), and medicines prescribed to patients outside of hospitals (too expensive for many pensioners). Compared to Japan, little has been done to adapt society and the economy to the emerging reality of an elderly population with improved capabilities. In sum, much remains to be accomplished in the period out to 2020 to ensure that the more dynamic newer cohorts of the elderly will be able to fulfil their potential and thereby make greater contributions to the economy and society.

Taking these circumstances into account, the Russian government should both adjust policies to acknowledge the enhanced capabilities of the elderly and make greater efforts to improve the income, enabling environment and medical care of older people. With respect to the former, the government would be justified in raising the low retirement ages of women (now 55 years) and men (60 years) to establish a more rational relationship between life expectancy and retirement (Eich et al. 2012). It could be productive to explore options for co-funding of pensions. Concerning the latter, the government could raise in real terms the pensions of the lower-income elderly and could do more to close the substantial gaps that exist between Russia and OECD countries in the areas of social care (e.g. increasing the numbers of social workers and places in high-quality residential homes for the elderly) and specialized medical care related to complicated non-communicable diseases (e.g. cardiovascular, diabetes, cancer and dementia). Overall, it is likely that reforms benefiting the elderly would generate net benefits to the economy and society in Russia.

## Appendix

Table 10

**Table 10:** Life experiences of the Russia birth cohort of 1930 turning 60 in 1990

**Category**	**Childhood (1–15): 1930–1944**	**Young adult (16–49): 1945–1979**	**Mature adult (50–59): 1980–1989**	**Early elderly (60–69): 1990–1999**
**Politics**	Communist USSR: 1930–1944 Stalin, class war, Great Terror, international tensions, World War II.	Communist USSR: 1945–1953 Stalin, victory of USSR in World War II, purges, start of Cold War; 1953–1957 Interregnum Malenkov; 1957–1964 Krushchev; 1964–1979 Brezhnev and mature socialism.	Communist USSR: 1980–82 Brezhnev; 1982–84 Andropov, 1984–85 Chernenko, 1985–89 Gorbachev and perestroika, political instability.	Communist USSR: 1990–91 late Gorbachev, collapse of communist system, break-up of USSR; Democratic Russia: 1992–99 Yeltsin, weak state, erratic leadership.
**Economy**	Command: 1930–40 FYPs, rapid industrialization, arms buildup, collectivization, shortage economy, famine, poverty; 1941–1944 war economy, subsistence living.	Command: 1945–53: war economy, famine, demobilization, reconstruction; 1953–64: rapid industrialization, rise in consumption; 1964–79: decelerating economic growth, arms buildup, improving living standards.	Command: 1980–84: stagnation (decelerating growth); 1985–89: acceleration strategy, 12th FYP, perestroika reforms, disruption of planning system.	Command: 1990–91: reform failures, disintegraion of USSR; Market: 1992–99 market reforms, failure of stabilization, high inflation, barter, voucher privatization, loss of savings, cuts in real wages, poverty.
**Society**	Socialist: 1930–40: rapid urbanization, rural life collapse, destruction of civil society, rising social stress; 1941–44 war, evacuations, acute stress.	Socialist: 1945–56: Rapid urbanization, weak civil society, atomization of family life, stress; 1957–79 stability, traditional social capital.	Socialist: 1980–84: social stability, traditional social capital; 1985–89: development of civil society, glasnost’, social instability.	Socialist: 1990–99: undermining of social beliefs, growth of civil society, weakening of social capital, intensification of social stresses.
**Health Environment**	Industrial pollution, unhygienic living conditions, mechanization, war, refugees, stress.	1945–46 war, refugees, stress; 1947–79 Industrial pollution, unhygienic living conditions, mechanization.	1980–89 Industrial pollution, unhygienic living conditions, mechanization.	1990–99: decline in pollution, growth of psycho-social stress.
**Health behaviours**	Poor diet, physical fitness in schools.	Poor diet, heavy alcohol consumption, smoking, inadequate exercise.	Poor diet, heavy alcohol consumption, smoking, inadequate exercise.	Poor diet, heavy alcohol consumption, smoking, inadequate exercise.

Sources: This table and the related text in section three are based on extensive research carried out by the author that involved examination of Soviet and Russia data and literature on the USSR and Russia by academics, governments and international organizations. Due to space constraints references are made primarily to the author’s lectures at Oxford University and publications (Davis 1983a, b, 1987, 1988, 1990, 1993, 1999, 2001a, 2002, 2006, 2014, 2015, 2016) that contain detailed citations of sources

Table 11

**Table 11:** Life experiences of the Russia birth cohort of 1935 turning 60 in 1995

**Category**	**Childhood (1–15): 1935–1949**	**Young adult (16–49): 1950–1984**	**Mature adult (50–59): 1985–1994**	**Early elderly (60–69): 1995–2004**
**Politics**	Communist USSR: 1935–1949 Stalin, class war, Great Terror, international tensions, World War II, victory of USSR, start of Cold War.	Communist USSR: 1950–1953 Stalin, purges, Cold War; 1953–1957 Interregnum Malenkov; 1957–1964 Krushchev; 1964–1979 Brezhnev and mature socialism; 1982–84 Andropov, Chenenko.	Communist USSR: 1985–91 Gorbachev and perestroika, political instability, collapse of communism, break-up of USSR; Democratic Russia: 1992–94 Yeltsin, weak state, erratic leadership.	Democratic Russia: Yeltsin 1995–1999, 1999–2004 Putin and strengthening of state, political stability.
**Economy**	Command: 1935–40: FYPs, industrialization, arms buildup, shortage economy, poverty; 1941–1945: war economy, subsistence living 1946–49: famine, demobilization, reconstruction.	Command: 1950–53: reconstruction of industry, austerity; 1953–64: re-orientation to light industry and agriculture, rise in consumption; 1964–84: decelerating economic growth, arms buildup, energy exports, improving living standards.	Command: 1985–91: acceleration, perestroika, disruption of planning, reform failures, disintegration of USSR; Market: 1992–94: market reforms, failure of stabilization, high inflation, barter, voucher privatization, loss of savings, cuts in real wages, poverty.	Market: 1995–99: market reforms, high inflation, output decline, virtual economy, corrupt privatization, wage arrears, poverty, financial crisis in 1998; 2000–04: import-substitution and energy export recovery, high growth of GDP and living standards.
**Society**	Socialist: 1935–40: rapid urbanization, rural life collapse, weak civil society, rising social problems; 1941–45 war, evacuations, social strains, orphans; 1946–49 difficulties in family life.	Socialist: 1950–56: urbanization, weak civil society, atomization of family life; 1957–84 relaxation of social controls, stability, traditional social capital.	Socialist: 1985–91: growth civil society, glasnost’, social instability Democratic: 1992–94: collapse of social beliefs, growth of civil society, weakening of social capital, intensification of social problems.	Democratic: 1995–99: social demoralization, growth of civil society, weakening of social capital, intensification of social problems; 2000–04 increased social stability and optimism.
**Health environment**	1935–49: Industrial pollution, unhygienic living conditions, mechanization, war, refugees, stress.	1950–84 Industrial pollution, improving living conditions, mechanization.	1985–91 Industrial pollution, improving living conditions, mechanization; 1992–94 decline in pollution, increase in stress.	1995–99: decline in pollution, growth of psycho-social stress; 2000–04 improvements in environment, reduction in stress.
**Health behaviours**	1935–49: Poor diet, physical fitness in schools.	1950–84: unbalanced but improving diet, heavy alcohol consumption, smoking, inadequate exercise.	1985–91: improving diet, reduced alcohol consumption, smoking, inadequate exercise; 1992–94 worse diet, more alcohol.	1995–2004: poverty-related poor diet, heavy alcohol consumption, smoking, inadequate exercise.

Sources: This table and the related text in section three are based on extensive research carried out by the author that involved examination of Soviet and Russia data and literature on the USSR and Russia by academics, governments and international organizations. Due to space constraints references are made primarily to the author’s lectures at Oxford University and publications (Davis 1983b, 1987, 1988, 1993, 1999, 2001a, 2002, 2006, 2014, 2015, 2016) that contain detailed citations of sources

Table 12

**Table 12:** Life experiences of the Russia birth cohort of 1940 turning 60 in 2000

**Category**	**Childhood (1–15): 1940–1954**	**Young adult (16–49): 1955–1989**	**Mature adult (50–59): 1990–1999**	**Early elderly (60–69): 2000–2009**
**Politics**	Communist USSR: 1940–53 Stalin, class war, Great Terror, international tensions, World War II, victory of USSR, start of Cold War; 1954 Interregnum Malenkov.	Communist USSR: 1955–1957 Interregnum Malenkov; 1957–1964 Krushchev; 1964–1982 Brezhnev and mature socialism; 1982–84 Andropov, Chernenko; 1985–89 Gorbachev and perestroika.	Communist USSR: 1990–91 Gorbachev, political instability, collapse of communism, break-up of USSR; Russia democracy: 1992–99 Yeltsin, weak state, erratic leadership; 1999 PM Putin.	Democratic Russia: 2000–2009 President Putin and strengthening of state, political stability, tightening of political controls.
**Economy**	Command: 1940: industrialization, arms buildup, poverty; 1941–1945: war economy, subsistence living; 1946–53: famine, demobilization, reconstruction; 1954 shift of priorities to consumers but poverty.	Command: 1955–64: attention to light industry and agriculture, rise in consumption; 1964–84: decelerating economic growth, arms buildup, energy exports, improving living standards; 1985–89 perestroika.	Command: 1990–91: perestroika, reform failures, disintegration of USSR; Market: 1992–99: high inflation, output decline, virtual economy, corrupt privatization, poverty, 1998 financial crisis.	Market: 2000–09: recovery based on import-substitution and energy exports, high growth of GDP and living standards, effective response by state to Global Financial Crisis.
**Society**	Socialist: 1940: urbanization, weak civil society, rising social stress; 1941–45 war, evacuations, acute stress, orphans; 1946–54 austerity, strains on family life.	Socialist: 1955–56: weak civil society, stress; 1957–84 relaxation of social controls, stability, traditional social capital; 1985–89: development of civil society, glasnost’, instability.	Socialist: Democratic: 1990–99: collapse of social beliefs, growth of civil society, weakening of social capital, intensification of social stresses.	Democratic: 2000–09 increased social stability and optimism, improvements of family situations, more NGOs and civil society, constraints on media.
**Health environment**	1940–45: war, refugees, stress 1946–54 industrial pollution, unhygienic living conditions, mechanization.	1955–84 Industrial pollution, improving living conditions, mechanization; 1985–89 reforms to improve environment.	1990–91 Soviet-era problems; 1992–99 declines in pollution, reduced safety, increase in stress.	2000–09 improvements in hygiene, reduced pollution, reduction in stress, protection against the GFC.
**Health behaviours**	1940–45: Subsistence diet, physical fitness in schools; 1946–54 austerity, poor diet.	1955–84: unbalanced but improving diet, heavy alcohol consumption, smoking, inadequate exercise; 1985–89: better diet, reduced alcohol consumption, smoking.	1990–91: improving diet, reduced alcohol, inadequate exercise; 1992–99 deterioration of diet, more alcohol and smoking, intravenous drug use, sharing needles.	2000–09: improving diet, moderation of alcohol consumption and smoking, healthier life style.

Sources: This table and the related text in section three are based on extensive research carried out by the author that involved examination of Soviet and Russia data and literature on the USSR and Russia by academics, governments and international organizations. Due to space constraints references are made primarily to the author’s lectures at Oxford University and publications (Davis 1983b, 1987, 1988, 1993, 1999, 2001a, 2002, 2006, 2014, 2015, 2016) that contain detailed citations of sources

Table 13

**Table 13:** Life experiences of the Russia birth cohort of 1950 turning 60 in 2010

**Category**	**Childhood (1–15): 1950–1964**	**Young adult (16–49): 1965–1999**	**Mature adult (50–59): 2000–2009**	**Early elderly (60–69): 2010–2019**
**Politics**	Communist USSR: 1950–53 Stalin, international tensions, Cold War; 1954 Interregnum Malenkov; 1955–1957 Interregnum Malenkov; 1957–1964 Khrushchev.	Communist USSR: 1965–1984 Brezhnev, Andropov, Chernenko; 1985–91 Gorbachev, perestroika, political instability, collapse of communism, break-up of USSR; Democratic Russia: 1992–99 Yeltsin, weak state, erratic leadership.	Democratic Russia: 2000–2009 President Putin and strengthening of state, political stability, tightening of political controls.	Democratic Russia: 2010–2012 PM Putin, 2012- President Putin, strong state, political stability, controls on media, 2013- Ukraine and Syria conflicts.
**Economy**	Command: 1950–53: reconstruction; 1953–64: re-orientation to light industry and agriculture, rise in consumption.	Command: 1965–84: slowing GDP growth, arms buildup, energy exports, rising consumption; 1985–91 perestroika, reform failures, disintegration USSR; Market: 1992–99: high inflation, output decline, corrupt privatization, poverty, 1998 financial crisis.	Market: 2000–09: recovery based on import-substitution and energy exports, high growth of GDP and living standards, effective response by state to Global Financial Crisis.	Market: 2010–13: post-GFC slow growth, rising consumption; 2014–19: negative/low growth, drop in energy prices, economic sanctions, import substitution, disruption of consumption growth.
**Society**	Socialist: 1950–53 austerity, strains on family life; 1954–64: weak civil society, relaxed social controls, stability, developed social capital.	Socialist: 1965–84: relaxation of social controls, stable social capital; 1985–89: growth of civil society, instability Democratic: 1990–99: growth of civil society, weakening of social capital, intensified social problems.	Democratic: 2000–09 increased social stability and optimism, improvements of family situations, more NGOs and civil society, constraints on media.	Democratic: 2010–19: social/family stability, decline of optimism, greater social problems related to lower living standards and higher unemployment.
**Health environment**	1950–64: industrial pollution, unhygienic living conditions, mechanization, stresses of shortage economy.	1965–91: social stresses, industrial pollution, better living conditions, more technology, environmental controls; 1992–99 lower pollution, reduced safety, increased stress.	2000–09 improvements in hygiene, reduced pollution, reduction in stress, protection against the GFC.	2010–19 improvements in hygiene, controls on pollution, rise in social stress due to economic instability.
**Health behaviours**	1950–53 austerity, poor diet; 1955–64: better diet, inadequate exercise, under-reporting of illnesses.	1965–91: improving diet, heavy alcohol consumption, smoking, inadequate exercise, hidden illness; 1992–99 deterioration of diet, more alcohol and smoking, intravenous drug use, sharing needles.	2000–09: improving diet, moderation of alcohol consumption and smoking, healthier life style.	2010–19: improving diet, reductions in alcohol consumption and smoking, healthier life style.

Sources: This table and the related text in section three are based on extensive research carried out by the author that involved examination of Soviet and Russia data and literature on the USSR and Russia by academics, governments and international organizations. Due to space constraints references are made primarily to the author’s lectures at Oxford University and publications (Davis 1983b, 1987, 1988, 1993, 1999, 2001a, 2002, 2006, 2014, 2015, 2016) that contain detailed citations of sources

Table 14

**Table 14:** Life experiences of the Russia birth cohort of 1960 turning 60 in 2020

**Category**	**Childhood (1–15): 1960–1974**	**Young adult (16–49): 1975–2009**	**Mature adult (50–59): 2010–2019**	**Early elderly (60–69): 2020–2029**
**Politics**	Communist USSR: 1960–1964 Khrushchev; 1965–1974 Brezhnev, Cold War, detente.	Communist USSR: 1975–1984 Brezhnev, Andropov, Chernenko; 1985–91 Gorbachev, collapse of communism and USSR; Democratic Russia: 1992–99 Yeltsin, weak state, 2000–2009 President/PM Putin, stronger state, political stability.	Democratic Russia: 2010–2012 PM Putin, 2012–19 President Putin, strong state, political stability, controls on media, 2013- Ukraine and Syria conflicts.	Democratic Russia: 2020–29 President Putin and successors, strong state, political stability, controls on media, international tensions.
**Economy**	Command: 1960–64: re-orientation to light industry and agriculture, rise in consumption; 1965–74: slowing GDP growth, arms buildup.	Command: 1975–84: slow growth, arms buildup, energy exports, rising consumption; 1985–91 perestroika, reform failures; Market: 1992–99: high inflation, output decline, corruption, poverty, 1998 crisis; 2000–09: recovery of GDP and consumption, GFC.	Market: 2010–13: post-GFC slow growth, rising consumption; 2014–19: negative/low growth, drop in energy prices, economic sanctions, import substitution, falling living standards.	Market: 2020–29: higher energy prices, intensified reforms, tight budget controls, slow growth of GDP and living standards.
**Society**	Socialist: 1960–74: weak civil society, relaxation of social controls, stability, traditional social capital.	Socialist: 1975–84: stable social capital; 1985–89: growth of civil society, instability; Democratic: 1990–99: growth of civil society, weakening of social capital; 2000–09 increased social stability and optimism, richer civil society, constraints on media.	Democratic: 2010–19: social stability, decline of optimism, family improvements, greater social problems due to to lower living standards and higher unemployment.	Democratic: 2020–29: social stability but less optimism, family improvements, social problems related to unemployment, inequality and poverty.
**Health environment**	1960–74: industrial pollution, unhygienic living conditions, more technology, stresses of shortages.	1975–91: industrial pollution, better living conditions, more technology; 1992–99 lower pollution, reduced safety, intense social stress; 2000–09 improved hygiene, reduced pollution and stress.	2010–13 improvements in hygiene, controls on pollution; 2014–19 worsening health environment due to budget cuts, greater social stress.	2020–29: growing economy results in improved hygiene, tighter controls on pollution, but greater social stress for low income groups.
**Health behaviours**	1960–74: improving diet, inadequate exercise, under-reporting of illnesses.	1975–91: improving diet, rise and decline of alcohol consumption, smoking, inadequate exercise, hidden illness; 1992–99 deterioration of diet, more alcohol and smoking, intravenous drug use, obesity; 2000–09: improving diet, moderation of alcohol consumption and smoking, healthier life style.	2010–19: healthier life style for middle classes (better diet, reduced alcohol consumption and smoking) but not for lower income groups (e.g. rising obesity).	2020–29: healthier life styles for most citizens in all social classes (reduced alcohol consumption, smoking and drugs), but obesity a major problem.

Sources: This table and the related text in section three are based on extensive research carried out by the author that involved examination of Soviet and Russia data and literature on the USSR and Russia by academics, governments and international organizations. Due to space constraints references are made primarily to the author’s lectures at Oxford University and publications (Davis 1983b, 1987, 1988, 1993, 1999, 2001a, 2002, 2006, 2014, 2015, 2016) that contain detailed citations of sources

Table 15

**Table 15:** Human Capabilities of the Elderly in Russia Index: Normalisation and Weights of Indicators

		Normalisation boundaries for adjusted indicators			Weights (1–10) for geometric mean
	Measure	Adjustment value	Minimum	Minimum	
**1. Education and employment (EW) indicator values**					
1.1 Educational Attainment of population aged 60–64	Higher & Intermediate per 1000	0	100	700	5
1.2 Working OAPs as share of total OAPs	%	0	10	50	3
1.3 OAP aged 60–72 as share of total employment	%	0	2	8	2
**2. Income security (IN) indicator values**					
2.1 Pension coverage	%	0	70	100	3
2.2 Old age pension as a share of the average wage	%	0	30	60	2
2.3 OAP as share of subsistence minimum	%	0	50	300	2
2.4 Real GDP per old age pensioner Index	1990 = 100	0	200	500	3
**3. Enabling environment (EE) indicator values**					
3.1 Social capital: Index of happiness Gen population	% On the Whole Happy	0	20	80	1
3.2 Social care: Residence beds	Number	0	200	300	2
3.3 Public safety: Reported crime of theft	Number	1600	100	700	2
3.4 Housing: Urban living space	Sq M per capita	0	10	30	2
3.5 Public transportation	Passenger KM-millions	0	400	800	2
3.6 Political participation: Share elderly voting x share voting for ruling party	%	0	30	90	1
**4. Medical care (MC) indicator values**					
4.1 Medical staff per elderly person	Number	0	60	120	3
4.2 Real health expenditure index	1990 = 100	0	50	200	3
4.3 Capacity of polyclinics	Visits per Shift	0	20	30	2
4.4 Medical operations in hospitals	Number	0	40	80	2
**5. Health status (HS) indicator values**					
5.1 Total population morbidity (Incidence) rate	Cases per 1000	900	60	300	1
5.2 Cardiovascular morbidity (Incidence) rate	Cases per 1000	30	1	20	2
5.3 Disability (Incidence)	Cases per 1000	12	2	8	1
5.4 Mortality rate of age group 60–64 years	Deaths per 1000	50	2	20	3
5.5 Life expectancy at 60	Years	0	10	20	3

(1) The values of five indicators related to crime, illness and mortality were subtracted from an adjustment factor so that increases in them would reflect improvements, (2) The normalisation was carried out on the adjusted values of indicators

## Footnotes

1 This study accepts the United Nations (UNFPA 2012, 20) boundary of 60 years for the category of elderly. Some developed countries, such as the USA, and international organizations prefer an old age boundary of 65 years (see World Bank 2007). Russian statistics often define the elderly as those at and above the official retirement ages, which are 60 years for men and 55 years for women.

2 The concept of ‘enabling environment’ of the elderly refers to the arrangements in a society that facilitate older persons ageing actively and continuing to participate in political, social, economic and cultural life (see UNFPA 2012 and HelpAge International 2013): maintaining supportive networks (social capital); guaranteeing medical and social care; protection against crime; provision of appropriate housing; availability of public transportation; and ability to participate in the political system.

3 Section 2 explains that the Capabilities of the elderly have five Components: education and work experience (EW); income security (IS); enabling environment (EE); medical care (MC); and health status (HS).

4 A cohort is a group of people who are defined by a common characteristic (year of birth in this article) and share similar experiences over a defined period (Bhopal 2012, 306–07). The cohort approach of this article was influenced by Engelaer (2013), which studied birth cohorts of the elderly in Japan.

5 In the appendix tables the Dimensions of experiences in the four phases of life mentioned above are: politics, economy, society, health environment and health behaviours.

6 Soviet scholars (Bednyy 1972, Valentei 1977, 1978) produced studies of the different life experiences of those born in the early years of the USSR from those born in the post-Stalin period. More recent scholars have used birth cohort methods in analyses of trends in mortality and life expectancy in Russia (e.g. Zakharov 1999; Shkolnikov et al. 2014).

7 The statistics cited in this section are from the statistical yearbooks of GKRFS, notably: *Demograficheskii Ezhegodnik Rossii* 2015, *Rossiiskii Statisticheskii Ezhegodnik* 2015, *Rossiya v Tsifrakh* 2015, *Sotsial’noe Polozhenie i Uroven Zhizni Naseleniya Rossii* 2015, and *Zdravookhranenie v Rossiiskoi Federatsii* 2015.

8 For example, UNPD (2010) projected a decline in the population of Russia to around 142 million in 2020.

9 According to United Nations Development Program (UNDP) (1990, 11) the concepts of capabilities provide the foundation of measures of Human Development: *Human development...brings together the production and distribution of commodities and the expansion and use of Capabilities. It also focuses on choices - on what people should have, be and do to be able to ensure their own livelihood.*

10 Health status (HS) is widely accepted as a component of human capital. An early work by Grossman (1999) stated that *…a person’s stock of knowledge affects his market and nonmarket productivity, while his stock of health determines the total amount of time he can spend producing money earnings and commodities.* More recently HelpAge International (2013) observed that …*in recent years, health (including physical capacities, cognitive function and mental health) has come to be seen as a fundamental component of human capital.*

11 Among other interesting and relevant works reviewed were Zahidi (2014) on a human capital index and Zaidi (2014) on an active ageing index.

12 The simple formula below for HS and IN is: $Dimension\  Sub- Index=\frac{Actual\  Value- Minimum\  Value}{Maximum\  Value- Minimum\  Value}$. A useful property of the geometric mean in calculating an aggregate index of mixed indicators is that 1% increases in two different indicators (say life expectancy and GDP per capita) will have the same impacts on the index.

13 According to UNDP (1990, pg. 11): *Theories of human capital formation…view human beings primarily as means rather than as ends. They are concerned only with the supply side - with human beings as instruments for furthering commodity production… But human beings are more than capital goods for commodity production. They are also the ultimate ends and beneficiaries of this process. Thus, the concept of human capital formation…captures only one side of human development, not its whole.*

14 Z-scores of a data point (*X*_*i*_) are measures of its relationship to the mean (μ), expressed in standard deviation units σ: $Z\  Score=\frac{X_i-\mu}{\sigma}$ (Wonnacott and Wonnacott 1990, Chapter 4).

15 The author would like to thank colleagues involved in the HelpAge International *Global AgeWatch Index* project for general advice and for providing him with the 2013 and 2014 all-country data sets and formulas, which helped him to develop the methodology for the calculations related to the *HCERI*.

16 Section two explains that the *HDI* and *GAWI* normalize indicator values by using empirically-revealed low (worst country) and high (best country) standards. The single-country *HCER Index* measures developments over time, not across the world, so international comparators are not available. The minimum and maximum levels were selected to reflect feasible low and high levels for specific indicators. The standards have reasonably generous tolerance limits because the normalised values they generate are weighted and included in a Component Sub-Index with other indicators, which then are combined into the Aggregate Index. So there is a low sensitivity of the overall index to possible errors in the setting of the low-high standards of any one indicator.

17 If all indicators in a category were of roughly the same importance they were given equal weights. If the author considered one or more indicators to be more significant for Human Capabilities in a Component (e.g. Life Expectancy at 60), then they were given greater integer values than the others, subject to the rule that the sum would be 10. The sensitivity of the overall index to these choices was low because the *HCERI* has five Components, numerous indicators, and a restricted range of weights.

18 The evaluations of life experiences and capabilities of cohorts in the sub-sections of section three are based on detailed examinations by the author of Soviet and Russian statistical sources and literature covering the period 1930–2016. The research by the author has been presented in lectures at Oxford University and in his numerous publications cited in the notes to the tables. Specific sources are mentioned in the text if they are of fundamental importance or were not mentioned in past works of the author.

19 The author has had close professional contacts over many years with Prof. Sergei Shishkin, who was the Head of the Working Group on Health for *Strategiya* 2020 and is playing the same role in the current work of Kudrin’s Centre for Strategic Research on *Strategiya Rossii na 2018-2024 gg*. Prof. Shishkin has provided the author with his assessment of progress in implementing the proposed measures of *Strategiya* *2020*, which makes it clear that significant successes have been achieved in altering the health-related behaviours of the population (smoking, consumption of alcohol and drugs), whereas not much has been accomplished in altering the structure and performance of medical establishments (polyclinics, hospitals).

20 Due to censorship, the USSR did not publish data related to most of the 22 indicators in the *HCERI* until the late 1980s in the *perestroika* period. As a result, it is not possible to calculate the *HCERI* for the period 1960–88. However, the generalizations in this section are based on substantial research on the Soviet period carried out by this author for both teaching and academic publications.

21 See and the comprehensive evaluation made by the Centre for Strategic Research of past programmes (including *Strategiya* 2020) made by (Dmitriev 2016) and new proposals made in *Strategiya Rossii na* 2018 *-2024 gg*.
